# A Near‐Infrared Fluorogenic Probe for Rapid, Specific, and Ultrasensitive Detection of Sphingosine in Living Cells and In Vivo

**DOI:** 10.1002/advs.202307598

**Published:** 2023-11-30

**Authors:** Yanyan Chen, Tingting Hao, Jing Wang, Yiming Chen, Xiuxiu Wang, Wei Wei, Jing Zhao, Yong Qian

**Affiliations:** ^1^ State Key Laboratory of Coordination Chemistry Chemistry and Biomedicine Innovation Center (ChemBIC) School of Chemistry and Chemical Engineering Nanjing University Nanjing 210023 China; ^2^ School of Engineering Vanderbilt University Nashville 37235 USA; ^3^ State Key Laboratory of Pharmaceutical Biotechnology School of Life Sciences Nanjing University Nanjing 210023 China; ^4^ Jiangsu Collaborative Innovation Center of Biomedical Functional Materials School of Chemistry and Materials Science Nanjing Normal University Nanjing 210023 China

**Keywords:** endogenous sphingosine imaging, fluorogenic probe, low background interference, rapid, specific and ultrasensitive detection, sphingosine detection

## Abstract

Sphingosine (Sph) plays important roles in various complex biological processes. Abnormalities in Sph metabolism can result in various diseases, including neurodegenerative disorders. However, due to the lack of rapid and accurate detection methods, understanding sph metabolic in related diseases is limited. Herein, a series of near‐infrared fluorogenic probes **DMS‐X** (X = 2F, F, Cl, Br, and I) are designed and synthesized. The fast oxazolidinone ring formation enables the **DMS‐2F** to detect Sph selectively and ultrasensitively, and the detection limit reaches 9.33 ± 0.41 nm. Moreover, it is demonstrated that **DMS‐2F** exhibited a dose‐ and time‐dependent response to Sph and can detect sph in living cells. Importantly, for the first time, the changes in Sph levels induced by Aβ_42_ oligomers and H_2_O_2_ are assessed through a fluorescent imaging approach, and further validated the physiological processes by which Aβ_42_ oligomers and reactive oxygen species (ROS)‐induce changes in intracellular Sph levels. Additionally, the distribution of Sph in living zebrafish is successfully mapped by in vivo imaging of a zebrafish model. This work provides a simple and efficient method for probing Sph in living cells and in vivo, which will facilitate investigation into the metabolic process of Sph and the connection between Sph and disease pathologies.

## Graphical abstract

A novel fluorogenic probe for specifical sphingosine detecting is constructed via a rational molecular design strategy. The probe could image Aβ_42_ oligomers‐induced Sph in neural cells and in vivo.

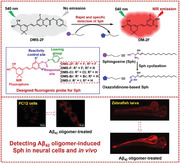

## 1 Introduction

Sphingolipids are structural molecules of cell membranes that play an important role in maintaining barrier function and fluidity. Sphingolipids were first isolated and identified from brain tissues in the 19th century by German biochemist Johann L. W. Thudichum, who named them after the Sphinx, a creature from Greek mythology, because of their enigmatic nature.^[^ ^2^ ^]^ Sphingolipid metabolites, ceramide (Cer), ceramide‐1‐phosphate (C1P), and sphingosine‐1‐phosphate (S1P) have been recognized as important signaling molecules that regulate cell growth, survival, immune cell trafficking, and vascular and epithelial cell integrity and are particularly important in inflammation and cancer.^[^ ^1^, ^3^ ^]^ In addition, sphingosine (Sph), another sphingolipid metabolite, constitutes a class of natural products containing a long aliphatic chain with a polar 2‐amino‐1,3‐diol terminus (2‐amino‐4‐trans‐octadecene‐1,3‐diol), which is generated from ceramides.^[^ ^4^ ^]^ It occurs in the cell membranes of all animals and many plants and plays an important role in a variety of complex biological processes, such as DNA damage,^[^ ^5^ ^]^ apoptosis,^[^ ^6^ ^]^ (including hippocampal neuron and astrocyte apoptosis),^[^ ^7^ ^]^ cell growth,^[^ ^8^ ^]^ differentiation, autophagic processes, and development.^[^ ^9^ ^]^ Significantly, abnormal sphingosine metabolism can induce various diseases, such as cancers and neurodegenerative diseases, including NPC (Niemann‐Pick disease type C), AD (Alzheimer's disease),^[^ ^10^ ^]^ and others.^[^ ^11^ ^]^ Based on the above, developing suitable probes or chemical tools to study sphingolipids and their metabolites and decipher their roles in cellular biology is urgently needed.

A plethora of approaches have been developed to detect cellular sphingolipids and metabolites. With respect to instrumental development, liquid chromatography‐mass spectrometry (LC‐MS), mass spectrometry (MS) and imaging mass spectrometry (IMS) techniques have become the method of choice for the detection and quantification of sphingolipid metabolites.^[^ ^12^ ^]^ Alternatively, sphingomyelin can be stained by fluorescent protein conjugates such as recombinant lysenin and equinatoxin.^[^ ^13^ ^]^ More interestingly and importantly, based on the widespread applications of fluorescence imaging technology in chemical biology, numerous methods for fluorescently labeling sphingolipids and metabolites have been developed and extensively used as sphingolipid probes to study the subcellular localization and metabolism of sphingolipids using fluorescence microscopy.^[^ ^14^ ^]^ Although there are many ways to label sphingolipids and metabolites, such as sphingosine, few methods have been developed for the direct detection of sphingolipids and metabolites, especially sphingosine.^[^ ^12^, ^13^, ^15^ ^]^ In 2020, Devaraj et al. first developed an ingenious fluorescently labeled aldehyde probe that chemoselectively reacts with terminal 1,2‐amino alcohol to detect endogenous Sph.^[^ ^16^ ^]^ Hence, the development of a simple and efficient novel method for highly selective and ultrasensitive detection of sphingosine in living cells remains highly useful and desirable.

**Scheme 1:**
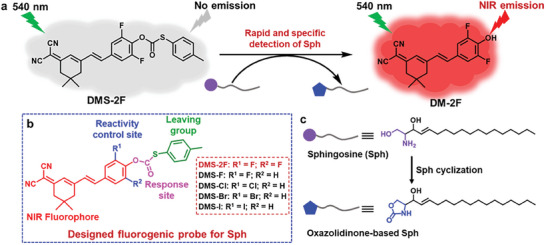
a) The response process of **DMS‐2F** with Sph; b) Designed structure of the fluorogenic probes **DMS‐X** and c) Structure of Sph and oxazolidinone‐based Sph after reacting with **DMS‐2F**.

In contrast to other sphingolipids, sphingosine has a unique terminal amino alcohol structure similar to that of norepinephrine (NE). Notably, Yin et al. pioneered the highly powerful “protect‐deprotect” strategy for NE detection.^[^ ^17^ **^]^** Herein, a series of fluorogenic probes, **DMS‐X** (X = 2F, F, Cl, Br, I), with thiocarbonate protecting groups were developed (**Scheme** **1**). Different halo substituents were introduced to control the reactivity between the probes and Sph. The results indicated that the 2,6‐difluoro‐substituted probe **DMS‐2F** can specifically and selectively detect sphingosine in a short time with good response sensitivity compared to other probes. Interestingly, neurotransmitters (NE, EP, and DA) did not interfere with the response of **DMS‐2F** to Sph. Furthermore, the results of intracellular studies suggest that **DMS‐2F** allows fluorescent imaging of exogenous and endogenous Sph, especially can monitor the Aβ_42_ oligomers‐induced Sph variation in live neutral cells and zebrafish in vivo. Based on the characteristics of reactive fluorescence probes, which largely avoid the background interference of probe molecules, this new NIR fluorogenic probe provides a new research idea for the selective and sensitive detection of sphingosine in vitro and in vivo.

## 2 Results and Discussion

The detailed synthesis procedure (Scheme S1, Supporting Information) and structural characterization (Figures S1–S30, Supporting Information) of fluorogenic probes **DMS‐X** are provided in the Supporting Information.

To evaluate the stability of **DMS‐X** (**DMS‐2F**, **DMS‐F**, **DMS‐Cl**, **DMS‐Br,** and **DMS‐I**), solutions of **DMS‐X** in PBS buffer were first exposed to a UV lamp (365 nm), and the change in fluorescence intensities of **DMS‐X** was monitored separately at different times. As shown in Figure S31a (Supporting Information), the fluorescence intensities of **DMS‐X** were almost unchanged after 52 h of exposure to a UV lamp. The investigation of fluorescent changes of **DMS‐X** in different pH buffers at different times also showed excellent stability (Figure S31b–f, Supporting Information). These results indicate that the **DMS‐X** probes have good photostability and acid‐base resistance properties.

**Figure 1:**
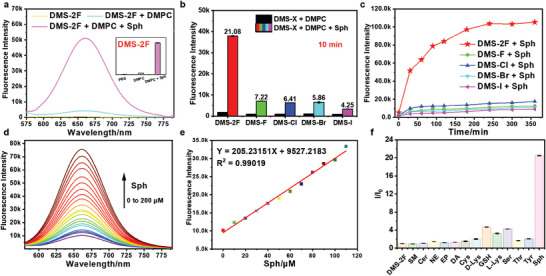
a) Fluorescence spectra of **DMS‐2F** in PBS and DMPC vesicles (with/without Sph), inset: histograms for fluorescent intensity at 660 nm; b,c) Fluorescence results of **DMS‐X** (**DMS‐2F**, **DMS‐F**, **DMS‐Cl**, **DMS‐Br** and **DMS‐I**) for Sph under physiological conditions and incubated for different times: b) 10 min, c) 0–360 min, respectively; d) Concentration response of **DMS‐2F** to Sph from 0 to 200 µm; e) Fluorescence spectrum linear range for Sph from 0 to 110 µm; f) Fluorescence intensity ratios of **DMS‐2F** and **DMS‐2F** toward various analytes (*λ*_ex_ = 540 nm, *λ*_em_ = 660 nm, slit = 10/10 nm, pH 7.4, 37 °C.

Next, the ability of **DMS‐X** probes (**DMS‐2F**, **DMS‐F**, **DMS‐Cl**, **DMS‐Br,** and **DMS‐I**) to react with Sph under physiological conditions (pH 7.4, 37 °C) was tested using the vesicles of DMPC to mimic biological membranes. After reacting with Sph, the fluorescent emission results showed that only **DMS‐2F** had significant fluorescence enhancement at 660 nm (**Figure** **1**a), while the other probes (**DMS‐F**, **DMS‐Cl**, **DMS‐Br,** and **DMS‐I**) exhibited only slight fluorescence changes (Figure S32a–h, Supporting Information). To further validate the superiority of **DMS‐2F** in detecting sphingosine, the fluorescence changes of **DMS‐X** were examined when incubated with Sph for different times in the DMPC system (with or without Sph). As shown in Figure 1b, an obvious fluorescence enhancement (I/I_0_ = 21.08) was detected in the **DMS‐2F** and Sph system when incubated at 37 °C for 10 min. With increasing incubation time, the fluorescence intensity of **DMS‐2F** gradually increased, reaching the highest intensity at 4 h (I/I_0_ = 57.60) (Figure 1c), which was fivefold faster than that reported for the Sph probe.^[^ ^16^ ^]^ However, the other probes (**DMS‐F**, **DMS‐Cl**, **DMS‐Br,** and **DMS‐I**) did not show significant fluorescence changes even after 6 h of incubation with Sph (Figure 1b; Figure S33, Supporting Information). These findings suggest that **DMS‐2F** can be used as a highly efficient fluorogenic probe in response to Sph.

To evaluate the appropriate pH range for the Sph recognition process of **DMS‐2F**, pH control experiments were carefully carried out in PBS‐buffered solutions (pH 3 to 9). After incubation with 200 µm Sph in buffer solutions of different pH values for 30 min at 37 °C, the fluorescence of **DMS‐2F** at 660 nm was significantly enhanced when the pH ranged from 6 to 9 (Figure S34, Supporting Information), indicating that **DMS‐2F** could efficiently fluorogenically detect Sph in the physiological pH range.

Concentration‐dependent fluorescence experiments were subsequently performed by incubating **DMS‐2F** (5 µm) with various concentrations of Sph (0–200 µm) in DMPC vesicles for 30 min at 37 °C. The results showed that the fluorescence intensity of **DMS‐2F** at 660 nm gradually increased with increasing Sph concentrations (Figure 1d). Moreover, an excellent linear relationship between the emission intensities was observed in the range of 0–110 µm (Figure 1e). The limit of detection (LOD) of **DMS‐2F** for Sph was calculated as 9.33 ± 0.41 nm according to the 3σ/m method. These findings suggest that **DMS‐2F** has the capability for ultrasensitive detection of Sph.

To further determine whether **DMS‐2F** could selectively detect Sph in living cells, **DMS‐2F** (5 εm) was incubated in DMPC vesicles composed of several naturally abundant lipid species and other possible interferents (including amino acids with structures similar to Sph, such as cysteine (Cys), lysine (Lys), serine (Ser), threonine (Thr), tyrosine (Tyr) (260 εm) and glutathione (GSH) (2 mm); naturally abundant lipid species, sphingomyelin (SM, 200 εm) and ceramide (Cer, 200 εm); and neurotransmitters: NE (norepinephrine, 200 εm), DA (dopamine, 200 εm), and EP (epinephrine, 200 εm) for 30 min at 37 °C. The results showed that a significant fluorescence turn‐on was observed when **DMS‐2F** (5 εm) was incubated with Sph (200 εm). However, when **DMS‐2F** was incubated with another analyte, there was a weak change in fluorescence intensity compared to the untreated **DMS‐2F** probe (Figure 1f). These results are consistent with the design of the **DMS‐2F** probe to selectively detect Sph in the DMPC system.

**Figure 2:**
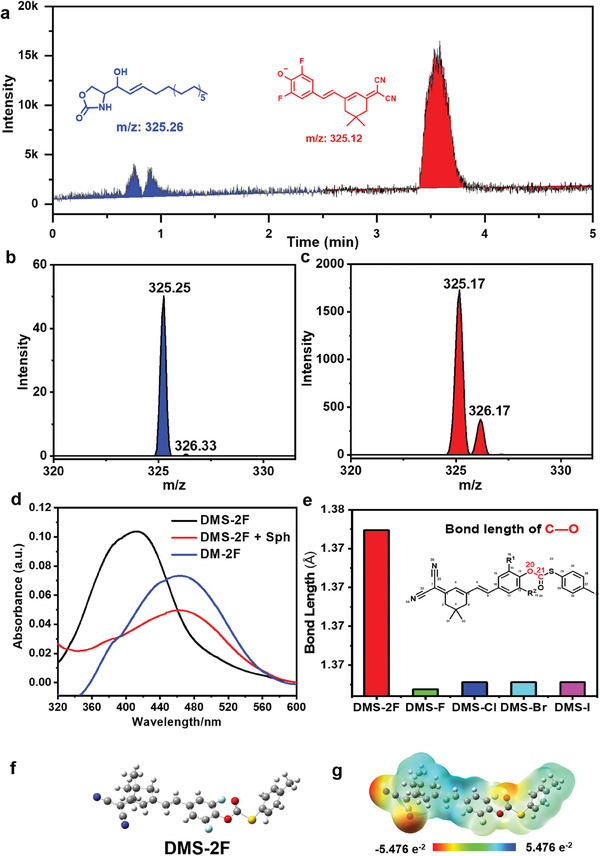
a,b,c) The LC‐MS of **DMS‐2F** (5 mm) in MeOH after reacting with Sph (100 mm) for 6 h at 37 °C; d) UV‒vis absorption spectra of **DM‐2F** (5 µm), **DMS‐2F** (5 µm) and **DMS‐2F** (5 µm) reacted with Sph (200 µm) in PBS buffer; e) Bond length between C^21^─O^20^ in **DMS‐X** evaluated by theoretical calculations, inset: the chemical structure of **DMS‐X**; f) View of the energy‐optimized structure and g) The molecular electrostatic potential (ESP) surface for **DMS‐2F** from Gauss View.

The specific response mechanism of **DMS‐2F** to Sph was confirmed by liquid chromatograph mass spectrometer (LC‐MS), high‐resolution mass spectrometry (HR‐MS), fluorescence spectroscopy, UV‐Visible spectroscopy, and theoretical calculation. **DMS‐2F** was exposed to Sph for 6 h at 37 °C, followed by LC−MS analysis and the reaction products of a five‐membered cyclic oxazolidinone‐based Sph compound (0.83 min) and the fluorophore **DM‐2F** (3.57 min) were successfully detected (**Figure** **2**a–c). In addition, HR‐MS experiments again confirmed that the fluorophore was released after **DMS‐2F** reacted with Sph, producing a five‐membered cyclic oxazolidinone‐based Sph compound (Figure S35, Supporting Information). Subsequently, the UV−vis and fluorescence response of **DMS‐2F** to Sph were determined by adding 200 εm Sph to 1 mL of PBS buffer containing 5 εm **DMS‐2F** and incubating at 37 °C for 6 h. As illustrated in Figure 2d, incubation with Sph resulted in a decrease in the UV‒vis absorption of **DMS‐2F** at 408 nm and an increase in absorption at 463 nm. The spectral properties of the system after reaction with Sph were in good agreement with the fluorophore **DM‐2F**. These results indicated that the response mechanism of **DMS‐2F** to Sph should be attributed to the nucleophilic addition and elimination reaction between ─NH_2_ of Sph and the thiocarbonate‐ester group of **DMS‐2F**. When the reaction is triggered, the fluorophore **DM‐2F** is released and emits significant fluorescence. The probable response mechanism of **DMS‐2F** to Sph is shown in Scheme 1. According to the proposed mechanism above, the bond cleavage between the carbon atom of the carbonyl group (C^20^) and the aryl oxide (O^21^) in probes is the key factor for the fluorogenic response of **DMS‐X** to Sph. Therefore, the bond energy of C^20^─O^21^ was a determining factor for screening the optimal probe for the response of Sph. To further understand the response priority of **DMS‐2F** and other probes (**DMS‐F**, **DMS‐Cl**, **DMS‐Br,** and **DMS‐I**) to Sph, theoretical calculations were carried out. The structures of **DMS‐X** were optimized by density functional theory (DFT) studies using the B3LYP 6–31G (d, p) levels (Figure 2f,g: Figure S36, Supporting Information), and the bond lengths of C^20^─O^21^ in **DMS‐X** were evaluated. As shown in Figure 2e, the bond length of **DMS‐2F** is significantly longer than that of the other probes, which further indicates that **DMS‐2F** has a better response effect than the other probes. Additionally, the ground‐state geometry structures of Sph and NE were optimized by DFT at the B3LYP/6‐31G levels to investigate the selective response mechanism of **DMS‐2F** toward Sph. The calculation results indicate that there are hydrogen bonding interactions between the amino group of NE and its neighboring hydroxyl group (Figure S37a, Supporting Information), which was detrimental to the **DMS‐2F** response to NE, whereas such interactions are absent in the structure of Sph (Figure S37b, Supporting Information). Additionally, the electrostatic surface potential (ESP) maps reveal that the amino moieties of Sph possess higher electronegativity compared to those of NE (Figures S37c,d, Supporting Information). These findings suggest that Sph can readily react with **DMS‐2F**, leading to its selective response.

These promising in vitro results prompted us to explore the imaging effect of **DMS‐X** in living cells. Using MTT assays, it was confirmed that **DMS‐X** was non‐toxic to different cell lines (Figures S38 and S39, Supporting Information). First, to determine whether **DMS‐2F** could react with Sph in live cells, A549 cells were incubated with **DMS‐2F** (10 εm) for 30 min, and then the cell medium was exchanged with a new medium containing 40 εm Sph and incubated for different amounts of time (30–150 min). As shown in Figure S40 (Supporting Information), as the Sph incubation time increased, the cells showed a gradual enhancement in red fluorescence emission within 1.5 h. To assess whether the response of **DMS‐2F** to Sph is concentration‐dependent in living cells, **DMS‐2F**‐pretreated A549 and HepG2 cells were incubated with different concentrations of Sph (0, 5, 10, and 20 εM). After 2 h of incubation, cells treated with exogenous Sph showed a dose‐dependent fluorescence enhancement signal (Figure S41, Supporting Information). Furthermore, to confirm that the observed increase in cellular fluorescence was a result of the release of the fluorophore **DM‐2F** after the reaction between **DMS‐2F** and Sph, A549 cells were further treated with **DM‐2F** (10 εm) (Figure S42, Supporting Information). Cells treated with **DM‐2F** exhibited a fluorescent imaging pattern consistent with that observed in cells treated with **DMS‐2F** and Sph. These results indicate that **DMS‐2F** could react with Sph in living cells within a short period of time and release an enhanced fluorescence signal by generating the fluorophore **DM‐2F** in a dose‐dependent manner.

Since elevated sphingosine in mammalian cells is closely associated with apoptosis, to evaluate the response ability of **DMS‐2F** to endogenous Sph levels in live cells, cancer cells (rat adrenal pheochromocytoma PC12 cells, human non‐small cell lung cancer A549 cells, and brain glioma U87 cell lines) and normal lung fibroblast MRC‐5 cells were exposed to serum‐free medium (opti‐MEM containing 10 εm **DMS‐2F**), which could result in elevated Sph levels. As shown in Figure S43a,b (Supporting Information), after 2 h of incubation, the fluorescence of live cells treated with opti‐MEM all showed a significant increase. Taken together, these results suggest that **DMS‐2F** is sensitive enough to probe native sphingosine in various live cells, and can be used for imaging differences in sphingosine levels in living cells.

**Figure 3:**
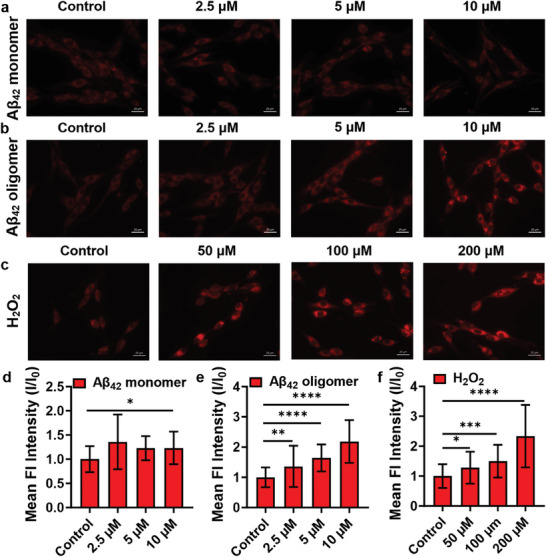
Fluorescence images of PC12 cells treated with different concentrations of a) Aβ_42_ monomers and b) Aβ_42_ oligomers for 12 h and then incubated with 10 εm **DMS‐2F** for 2 h. c) Fluorescence images of PC12 cells treated with different concentrations of H_2_O_2_ for 4 h and then incubated with 10 µm **DMS‐2F** for 2 h. d,e,f) Mean fluorescent intensities of PC12 cells in panels (a), (b), and (c), respectively. Scale bar: 20 µm. The data are expressed as the mean ± SD. One‐way ANOVA was used to compare multiple groups: ^*^ *P* ≤0.05, ^**^ *P* ≤0.01, ^***^ *P* ≤0.001, ^****^ *P* ≤0.0001.

Abnormal sphingolipid metabolism has been reported to induce Alzheimer's disease (AD), and substantial evidence also support that amyloid‐β (Aβ_42_) plays an important role in AD. In vitro, Aβ_42_ has been shown to induce apoptosis via the sphingomyelin pathway in various brain cells, including PC12 cells, etc.^[^ ^10^, ^18^ ^]^ Accordingly, we attempted to assess whether the level of Sph will be changed in PC12 cells after treatment with Aβ_42_ peptide. We first treated PC12 cells with 10 εm Aβ_42_ oligomers for different times and then incubated them with **DMS‐2F**, as shown in Figure S44 (Supporting Information), the fluorescence of PC12 cells treated with Aβ_42_ oligomers was significantly increased. These observations undoubtedly revealed that intracellular Sph was up‐regulated when neuronal cells were challenged by Aβ_42_ oligomers. To further correlate Sph generation with Aβ_42_ oligomers, PC12 cells were treated with different concentrations of Aβ_42_ oligomers and Aβ_42_ monomers. After 12 h of incubation, a gradual increase in fluorescence was observed in PC12 cells treated with Aβ_42_ oligomers, however, weak fluorescence changes were observed in Aβ_42_ monomers treated cells (**Figure** **3**a,b,d,e). Together, these findings suggested that neuronal cells experienced overexpression of Sph during Aβ_42_ administration and that the up‐regulated Sph levels correlated with both the incubation time and dosage of Aβ_42_ oligomers, indicating that the abnormal sphingolipid metabolism in neuronal cells is tightly related to the stimulation of Aβ_42_ oligomers.

Since the overexpression of Aβ protein induces mitochondrial oxidative stress and activates the intrinsic apoptotic cascade, and previous studies have illustrated that H_2_O_2_ could induce neurotoxicity in PC12 cells,^[^ ^18^, ^19^ ^]^ we further investigated the Sph variations induced by H_2_O_2_. PC12 cells were treated with different concentrations of H_2_O_2_, after 4 h of incubation, a gradual increase in fluorescence was detected in PC12 cells treated with H_2_O_2_ (Figure 3c,f), suggesting that the Sph variation occurred during the oxidative stress process. In addition, the changes of Sph levels after being treated with Aβ_42_ oligomers and H_2_O_2_ in other cell lines were also assessed. U87, A549, HepG2, and MRC5 cells were treated with Aβ_42_ oligomers and H_2_O_2_ for 12 and 4 h, respectively, and then incubated with **DMS‐2F** for 2 h. As shown in Figure S45 (Supporting Information), significant fluorescence enhancement was exhibited in PC12 and U87 cells, while the fluorescence in other cell lines was not evident. These results indicate that the fluorogenic probe **DMS‐2F** can successfully monitor the level changes of Sph during Aβ_42_ oligomers and H_2_O_2_‐induced apoptosis in neuronal cells.

**Figure 4:**
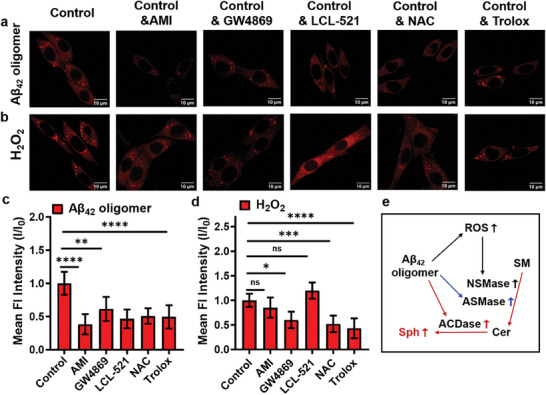
Fluorescence images of PC12 cells preincubated with different inhibitors and then treated with a) 10 εm Aβ_42_ oligomers for 12 h and b) 50 εm H_2_O_2_ for 4 h, and then incubated with 10 εm **DMS‐2F** for 2 h, scale bar: 10 µm; c,d) Mean fluorescent intensities of PC12 cells in panels (a) and (b), respectively; e) Schematic pathway proposed for the Aβ_42_ oligomers, ROS, and sphingolipid changes in the PC12 cells. All values are expressed as the mean ± SD of triplicates. One‐way ANOVA was used to compare multiple groups: ns *P*> 0.05, ^*^ *P* ≤0.05, ^**^ *P* ≤0.01, ^***^ *P* ≤0.001, ^****^ *P* ≤0.0001.

Since changes in Sph levels are associated with the abnormal sphingolipid metabolism in cells, to investigate the possible mechanisms of Aβ_42_ oligomers and H_2_O_2_‐induced changes in Sph levels, we further investigated intracellular sphingomyelinase and ceramidase activities in Aβ_42_ oligomers and H_2_O_2_ treated cells. PC12 cells were preincubated for 2 h in the presence of 10 µm amitriptyline hydrochloride (AMI, an acid sphingomyelinase inhibitor^[^ ^20^ ^]^), 20 µm GW4869 (a neutral sphingomyelinase inhibitor^[^ ^21^ ^]^), 10 µm LCL‐521 (an acid ceramidase inhibitor^[^ ^22^ ^]^), 500 µm N‐acetyl‐cysteine (NAC, an efficient antioxidant^[^ ^23^ ^]^) and 1 mm Trolox (a reactive oxygen scavenger ^[^ ^24^ ^]^), and then treated with Aβ_42_ oligomers (10 µm, 12 h) or H_2_O_2_ (50 µm, 4 h), respectively. As shown in **Figure** **4**a,c, the fluorescence of the cells preincubated with the inhibitors prior to the treatment with Aβ_42_ oligomers was lower than that of the control group. This suggests that the Aβ_42_ oligomers increase Sph levels by elevating intracellular ROS and enhancing intracellular acidic and neutral sphingomyelinase and acidic ceramidase activities. However, the increase in intracellular Sph levels in H_2_O_2_‐treated cells appeared to be more correlated with neutral sphingomyelinase activity. This is supported by the reduced imaging intensity observed in cells pretreated with the nerve sphingomyelinase inhibitor, GW4869, as well as the reactive oxygen scavengers, NAC and Trolox (Figure 4b,d). Therefore, it is hypothesized that the possible mechanism of Aβ_42_ oligomers and H_2_O_2_‐induced up‐regulation of intracellular Sph levels is as follows: Aβ_42_ oligomers induced an increase in intracellular ROS levels, which then activated neutral sphingomyelinase (NSMase), leading to the hydrolysis of sphingomyelin (SM) and the production of ceramides (Cer). This process ultimately results in an increase in intracellular Sph levels. Moreover, Aβ_42_ oligomers can activate acidic sphingomyelinase (ASMase) and acidic ceramidase (ACDase), which also contribute to the hydrolysis of SM and Cer, thereby increasing Sph levels (Figure 4e).

**Figure 5:**
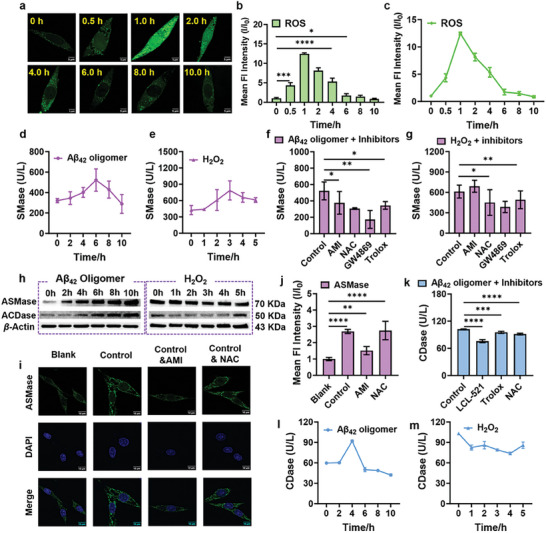
a) Fluorescence image of ROS in PC12 cells induced by Aβ_42_ oligomers at different time, scale bar: 5 µm; b,c) Mean fluorescent intensities of PC12 cells in panels (a); d,e). The level of SMase released in the supernatant of PC12 cells treated with Aβ_42_ oligomers and H_2_O_2_ for different durations measured by ELISA; f,g). The level of SMase released in the supernatant of PC12 cells pretreated with different inhibitors and then incubated by Aβ_42_ oligomers and H_2_O_2_ measured by ELISA; h) WB blot analysis of the expression of ASMase and ACDase in PC12 cells after incubation with Aβ_42_ oligomers and H_2_O_2_ at different times; i) Immunofluorescence analysis of ASMase in Aβ_42_ oligomers‐incubated PC12 cells pretreated or not pretreated with inhibitors AMI and NAC, scale bar: 10 µm; j) Mean fluorescent intensities of PC12 cells in panels Figure 5(h); k) The level of CDase released in the supernatant of PC12 cells pretreated with different inhibitors and then incubated by Aβ_42_ oligomers measured by ELISA; l,m). The level of CDase released in the supernatant of PC12 cells treated with Aβ_42_ oligomers and H_2_O_2_ for different durations measured by ELISA; All values are expressed as the mean ± SD of triplicates. One‐way ANOVA was used to compare multiple groups: ^*^ *P* ≤0.05, ^**^ *P* ≤0.01, ^***^ *P* ≤0.001, ^****^ *P* ≤0.0001.

To further validate the possible mechanisms proposed above, changes in ROS levels after different times of treatment of PC12 cells with Aβ_42_ oligomers were monitored using a ROS specific probe. We found that changes of ROS levels in cellular mitochondria upon Aβ_42_ oligomers treatment showed a gradual increase and reached the peak at 1 h. With prolonged treatment time, the ROS levels gradually decreased and eventually returned to the initial level (**Figure** **5**a–c; Figure S46, Supporting Information). The activations of SMase and CDase were then investigated. The treatment of PC12 cells with 1 µm Aβ_42_ oligomers and 50 µm H_2_O_2_ induced a time‐dependent increase in SMase activity and reached maximum levels at 6 and 3 h, respectively (Figure 5d,e; Figure S47, Supporting Information). Notably, the widely used ASMase and NSMase inhibitors AMI and GW4869 strongly inhibited Aβ_42_ oligomers‐induced SMase activation, respectively, while only GW4869 inhibited H_2_O_2_‐induced SMase activation, and the ROS scavenger NAC and Trolox inhibited both the Aβ_42_ oligomers‐ and H_2_O_2_‐induced SMase activation (Figure 5f,g). Immunoblotting and immunofluorescence experiments provided additional support for the aforementioned experimental results (Figure 5h–j; Figure S48, Supporting Information). This suggests that Aβ_42_ oligomers can induce the activation of ASMase and NSMase, respectively, whereas H_2_O_2_ can only induce the activation of NSMase. In addition, the ELISA test results for CDase indicated that the intracellular ceramidase level reached a maximum after 4 h of treatment with Aβ_42_ oligomers. It was observed that H_2_O_2_ did not activate intracellular CDase, but had an inhibitory effect, which aligns with the findings reported in the literature,^[^ ^25^ ^]^ (Figure 5l,m; Figure S49, Supporting Information). Moreover, the activation effect of ROS scavenger to Aβ_42_ oligomers induced CDase activity and immunoblotting experiments on ACDase further supported that Aβ_42_ oligomers can activate ACDase, while H_2_O_2_ inhibits its activity (Figure 5h,k; Figure S50, Supporting Information). Taken together, these results suggest that Aβ_42_ oligomers directly activate ASMase and ACDase, and also increase ROS levels, which activate NSMase and ultimately accelerate sphingolipid metabolism and increase the level of Sph.

**Figure 6:**
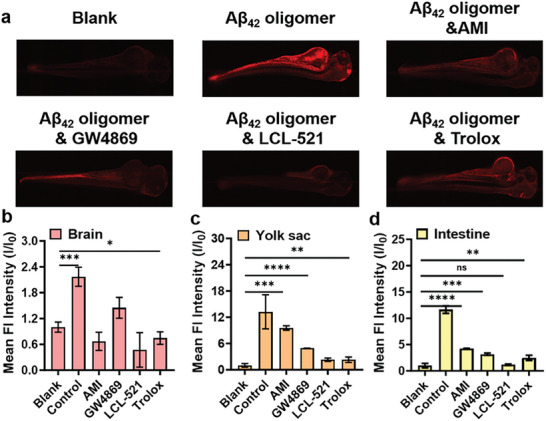
a) Fluorescence images of zebrafish preincubated with/without different inhibitors (20 µm AMI, 40 µm GW4869, 20 µm LCL‐521, and 1mm Trolox) and treated with 10 εm Aβ_42_ oligomers for 12 h and then incubated with 10 εm **DMS‐2F** for 2 h, scale bar: 200 µm; Mean fluorescent intensities of zebrafish position b) brain, c) yolk sac, and d) intestine, respectively. All values are expressed as the mean ± SD of triplicates. One‐way ANOVA was used to compare multiple groups: ns *P*> 0.05, ^*^ *P* ≤0.05, ^**^ *P* ≤0.01, ^***^ *P* ≤0.001, ^****^ *P* ≤0.0001.

Encouraged by the excellent results of live cell imaging, we further investigated the in vivo detection in a living animal model. The transparent zebrafish larva was selected as a model because zebrafish are optically transparent and have a high degree of homology with mammals.^[^ ^26^ ^]^ Zebrafish larvae (3 days old) were incubated with Aβ_42_ oligomers (10 εm) for 12 h and then treated with **DMS‐2F** (10 εm) for 2 h. As shown in **Figure** **6**, zebrafish treated with **DMS‐2F** without Aβ_42_ oligomers exhibited weak fluorescence, and notably, the Aβ_42_ oligomers treated zebrafish showed bright red fluorescence in the brain, yolk sac, and intestine. However, the fluorescence intensity of zebrafish pretreated with inhibitors (AMI, GW4869, LCL‐521, and Trolox) and further treated with Aβ_42_ oligomers and **DMS‐2F** was significantly weaker than that of zebrafish treated without inhibitors. Based on these results, it is shown that in vivo, Aβ_42_ oligomers can still induce the upregulation of Sph levels by the same mechanism as in neuronal cells. Hence, this new probe **DMS‐2F** successfully realized the detection of Sph in vivo.

## 3 Conclusion

In summary, we have developed a series of easily synthesized fluorogenic probes, named **DMS‐X**, by introducing different halogen substituents and thiocarbonate protecting groups. **DMS‐2F** can specifically detect sphingosine in a short period of time (within 4 h) and has excellent response sensitivity (LOD, 9.33 nm). **DMS‐2F** is capable of fluorescence tracing and imaging of exogenous and endogenous sphingosine in living cells. Importantly, we, for the first time, evaluated the Sph levels during Aβ_42_ oligomers and H_2_O_2_‐induced apoptosis of PC12 cells by fluorescent imaging approach and further verified that Aβ_42_ oligomers can induce abnormal sphingolipid metabolism by increasing the intracellular ROS levels and inducing the activation of sphingomyelinase and ceramidase during sphingolipid metabolism, which ultimately led to the increase of Sph level. Finally, we successfully realized the in vivo detection of Aβ_42_ oligomers‐induced Sph in a living zebrafish model. Based on the properties of reactive fluorogenic probes, this new probe greatly avoids the background interference of probe molecules and provides a novel strategy for monitoring sphingosine in living cells and in vivo.

## Conflict of Interest

The authors declare no conflict of interest.

## Supporting information

Supporting Information

## Data Availability

The data that support the findings of this study are available in the supplementary material of this article.
