# Supplementary material for: A Near‐Infrared Fluorogenic Probe for Rapid, Specific, and Ultrasensitive Detection of Sphingosine in Living Cells and In Vivo
Source: Adv Sci (Weinh). 2023 Nov 30;11(2):2307598. doi: 10.1002/advs.202307598 (PMC10787105; doi:10.1002/advs.202307598)
Supplement: Supplementary file 1 — Supporting Information [file ADVS-11-2307598-s001.pdf]

ADVANCED SCIENCE
Open Access

Supporting Information

for *Adv. Sci.*, DOI 10.1002/advs.202307598

A Near-Infrared Fluorogenic Probe for Rapid, Specific, and Ultrasensitive Detection of Sphingosine in Living Cells and In Vivo

Yanyan Chen, Tingting Hao, Jing Wang, Yiming Chen, Xiuxiu Wang, Wei Wei, Jing Zhao* and Yong Qian**

Supporting Information
©Wiley-VCH 2021
69451 Weinheim, Germany

A near-infrared fluorogenic probe for rapid, specific and ultra-sensitive detection of sphingosine in living cells and in vivo

Yanyan Chen,[a] Tingting Hao,[a] Jing Wang,[a] Yiming Chen,[d] Xiuxiu Wang,[a] Wei Wei,*[c] Jing Zhao,*[a] Yong Qian*[b]

Table of Contents

Table of Contents 2

Experimental Procedures 3

1. General Information 3

2. Experimental synthetic methods 3

3. Measurement of spectroscopic properties 4

3.1 Fluorescence measurements in DMPC vesicles 4

3.1.1 Time responses of **DMS-X** to Sphinganine (Sph) 4

3.1.2 Fluorescence responses of **DMS-2F** to Sph under different pH buffers 4

3.1.3 Fluorescence spectra of **DMS-2F** within different analyte 4

3.1.4 Fluorescent spectra of **DMS-2F** with different concentrations of Sph and the limit detection of **DMS-2F** to sph 4

4. Cell culture 4

5. MTT assay 4

6. Fluorescent imaging studies 4

6.1 Time-response of **DMS-2F** to Sph in living cells 4

6.2 Dose-response of **DMS-2F** to Sph in living cells 4

6.3 Detection of endogenous Sph in living cells 5

6.4 Detection of endogenous Sph variations induced by $A\beta_{42}$ monomer and $A\beta_{42}$ oligomers in PC12 cells ... 5

6.5 Detection of endogenous Sph variations induced by H_2O_2 in PC12 cells 5

6.6 Detection of endogenous Sph variations induced by $A\beta_{42}$ oligomers and H_2O_2 in different cell lines 5

6.7 Detection of Sph variations induced by Aβ42 oligomers and H_2O_2 under the preincubated of different inhibitors in PC12cells 5
7. ROS detection. 5
8. Western blot analysis. 5
9. Immunocytochemistry. 5
10. Live Imaging of Zebrafish. 5
Results and Discussion 5
Reference 30
Author Contributions 30

Experimental Procedures

1. General Information

All commercially available compounds were used as provided without further purifications. Chemicals and solvents were purchased from Sigma Aldrich, Aladdin, Bidepharm etc. TLC analysis was performed on a silica gel plate and chromatographic purification of products was performed on silica gel (200-300 mesh). ^{1}H and ^{13}C NMR data were acquired on a 400 MHz Bruker Avance NMR spectrometer (Germany), using DMSO-d_6 as solvent. Data are given in the following order: chemical shift (δ) values are reported in ppm with the solvent resonance as internal standard (DMSO- d_6: δ = 2.50 ppm for ^{1}H, δ = 39.52 ppm for ^{13}C); multiplicities are indicated as: s(singlet), d (doublet), dd (double doublet), t (triplet), q (quartet), m (multiplet); coupling constant values are given in Hertz (Hz). HRMS were performed on a Q Exactive mass spectrometer (Thermo Fisher, Germany) equipped with ESI interface and ion trap analyzer. Fluorescence spectra were recorded on a Shimadzu RF-6000 spectrofluorophotometer. Ultraviolet-visible (UV-vis) spectra were recorded on a Shimadzu UV-2550 spectrometer.

2. Experimental synthetic methods

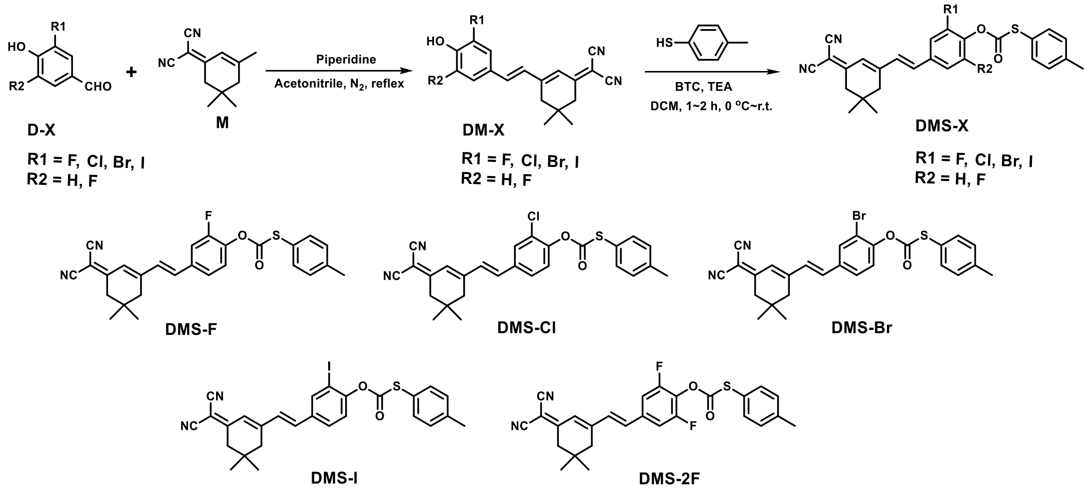

Scheme S1. General synthesis rule of **DMS-X.**

Synthesis and characterization：

DM-X: According to the reported synthesized methods,[1] compounds **DM-X** (X = 2F, F, Cl, Br and I) were synthesized. Compound **M** (1.1 equiv) and the aldehydes (compound **D-X**, 1 equiv) were dissolved in 20 mL acetonitrile with the addition of piperidine (three drops). The mixture was refluxed for 5 h. Then, the solvent was evaporated followed by the addition of 50 mL dichloromethane. Subsequently, the mixture was washed with water three times, respectively. The obtained organic phase was dried over anhydrous magnesium sulfate. Finally, the solvent was evaporated and the residue was purified by silica gel column chromatography to obtain the pure fluorophore as orange solids.

DM-2F: 1 mmol, 0.16 g, 48.5 %, ^{1}H NMR (400 MHz, DMSO-d_6, 298 K) δ 10.70 (s, 1H), 7.45 (dd, *J1* = 1.2 Hz, *J2* = 8 Hz, 2H), 7.35 (d, *J* = 16 Hz, 1H), 7.17 (d, *J* = 16 Hz, 1H), 6.85 (s, 1H), 2.61 (s, 2H), 2.50 (s, 2H), 1.01 (s, 6H); ^{13}C NMR (150 MHz, DMSO-*d6*, 298 K) δ 170.69, 156.00, 154.00, 153.92, 151.59, 151.52, 136.14, 135.65, 135.48, 135.32, 129.75, 127.60, 127.51, 127.43, 123.19, 114.30, 113.51, 111.83, 111.76, 111.68, 111.61, 76.80, 42.71, 38.61, 32.13. 27.90; HRMS: calculated to be [M-H]$^-$ 325.1152, found: 325.1159.

DM-F: 1 mmol, 0.14 g, 45.2 %, ^{1}H NMR (400 MHz, DMSO-*d6*, 298 K) δ 10.39 (s, 1H),, 7.60 (dd, *J1* = 2 Hz, *J2* = 12 Hz, 2H), 7.32 (dd, *J1* = 4 Hz, *J2* = 12 Hz, 1H), 7.23 (q, *J* = 16 Hz, 2H), 6.96 (t, *J* = 8 Hz, 1H), 6.83 (s, 1H), 2.60 (s, 2H), 2.51 (s, 2H), 1.01 (s, 6H); ^{13}C NMR (150 MHz, DMSO-d_6, 298 K) δ 170.70, 155.69, 152.89, 150.49, 147.20, 147.08, 137.42, 128.56. 128.50, 128.24, 126.07, 126.05, 122.51, 118.37, 118.33, 115.30, 115.11, 114.44, 113.64, 76.04, 42.75, 38.62, 32.12, 27.90; HRMS: calculated to be [M-H]$^-$ 307.1247, found: 307.1253.

DM-Cl: 1 mmol, 0.23g, 71.9 %, ^{1}H NMR (400 MHz, DMSO-d_6, 298 K) δ 10.70 (s, 1H),, 7.77 (d, *J* = 4 Hz, 1H), 7.48 (dd, *J1* = 2 Hz, *J2* = 8 Hz, 1H), 7.25 (dd, *J1* = 16 Hz, *J2* = 28 Hz, , 2H), 6.98 (d, *J* = 8 Hz, 1H), 6.84 (s, 1H), 2.60 (s, 2H), 2.51 (s, 2H), 1.01 (s, 6H); ^{13}C NMR (150 MHz, DMSO-d_6, 298 K) δ 170.73, 156.64, 154.96, 137.02, 129.56, 128.99, 128.88, 128.22, 122.56, 120.98, 117.32, 114.45, 113.63, 76.04, 42.75, 38.61, 32.12. 27.91; HRMS: calculated to be [M-H]$^-$: 323.0951, found: 323.0958.

DM-Br: 1 mmol, 0.31 g, 83.8 %, ^{1}H NMR (400 MHz, DMSO-d_6, 298 K) δ 10.77 (s, 1H),, 7.93 (d, *J* = 2 Hz, 1H), 7.52 (dd, *J1* = 2 Hz, *J2* = 8 Hz, 1H), 7.25 (dd, *J1* = 16 Hz, *J2* = 28 Hz, 2H), 6.96 (d, *J* = 8 Hz, 1H), 6.84 (s, 1H), 2.60 (s, 2H), 2.51 (s, 2H), 1.01 (s, 6H); ^{13}C NMR (150 MHz, DMSO-d_6, 298 K) δ 170.73, 156.67, 155.99, 136.93, 132.65, 129.47, 129.40, 128.16, 122.54, 117.01, 114.47, 11364, 110.63, 76.00, 42.75, 38.61, 32.12, 27.92; HRMS: calculated to be [M-H]$^-$ 367.0446, found: 367.0357.

DM-I: 1 mmol, 0.13 g, 31 %, ^{1}H NMR (400 MHz, DMSO-d_6, 298 K) δ 10.83 (s, 1H),, 8.10 (d, *J* = 2 Hz, 1H), 7.54 (dd, *J1* = 2 Hz, *J2* = 8 Hz,1H), 7.22 (dd, *J1* = 16 Hz, *J2* = 24 Hz, 2H), 6.89 (d, *J* = 8 Hz, 1H), 6.84 (s, 1H), 2.60 (s, 2H), 2.51 (s, 2H), 1.01 (s, 6H); ^{13}C NMR (150 MHz, DMSO-d_6, 298 K) δ 170.75, 158.60, 156.81, 138.83, 136.93, 130.20, 129.85, 127.86, 122.41, 115.60, 114.50, 113.67, 85.98, 75.84, 42.77, 38.62, 32.12, 27.92; HRMS: calculated to be [M-H]$^-$ 415.0307, found: 415.0302.

DMS-X: To a solution of p-toluenethiol (12 equiv.) and triphosgene (4 equiv.) in CH_2Cl_2 (10 mL) at 0 ℃ under N_2 atmosphere, after 10 min, triethylamine (12 equiv. in 5 mL of CH_2Cl_2) was added dropwise. The mixture was stirred for 10 min at 0 ℃, and then **DM-X** (1 equiv. in 5 mL of CH_2Cl_2) was added dropwise and finished within 10 min. All of the mixture was stirred for 10~20 min at 0 ℃. After the reaction was finished, the mixture was washed by water three times, respectively. The obtained organic phase was dried over anhydrous magnesium sulfate. Finally, the solvent was evaporated and the residue was purified by silica gel column chromatography to obtain yellow solid.

DMS-2F: 0.2 mmol, 76 mg, 79.7 %, ^{1}H NMR (400 MHz, DMSO-d_6, 298 K) δ 7.68 (d, *J* = 8 Hz, 2H),, 7.54 (m, 3H), 7.34 (d, *J* = 4 Hz, 2H), 7.22 (d, *J* = 16 Hz, 1H), 6.92 (s, 1H), 2.63 (s, 2H), 2.50 (s, 2H), 2.36 (s, 3H), 2.01 (s, 6H); ^{13}C NMR (150 MHz, DMSO-d_6, 298 K) δ 170.58, 167.91, 156.01, 154.91, 153.58, 153.53, 141.33, 137.17, 137.08, 136.99, 135.30, 134.21, 133.22, 130.91, 124.80, 122.13, 114.02, 113.25, 112.03, 111.81, 78.40, 42.66, 38.57, 32.14, 27.88, 21.31; HRMS: calculated to be [M-H]$^-$ 475.1286, found: 475.1292.

DMS-F: 0.2 mmol, 30 mg, 32.7 %, ^{1}H NMR (400 MHz, DMSO-d_6, 298 K) δ 7.82 (dd, *J1* = 4 Hz, *J2* = 12 Hz, 1H), 7.52 (m, 5H), 7.33 (d, *J* = 4 Hz, 2H), 7.27 (d, *J* = 20 Hz, 1H), 6.91 (s, 1H), 2.63 (s, 2H), 2.36 (s, 3H), 1.02 (s, 6H); ^{13}C NMR (150 MHz, DMSO-d_6, 298 K) δ 170.70, 168.14, 155.50, 155.11, 152.65, 141.02, 138.68, 138.54, 137.14, 137.07, 135.36, 135.23, 131,97, 130.79, 125.53, 124.82, 124.18, 122.72, 115.49, 115.30, 114.14, 113.34, 77.78, 42.78, 42.71, 38.59, 32.14, 27.89, 21.30; HRMS: calculated to be [M-H]$^-$ 415.1381, found: 457.1393.

DMS-Cl: 0.2 mmol, 52 mg, 54.7 %, ^{1}H NMR (400 MHz, DMSO-d_6, 298 K) δ 8.01 (d, *J* = 1.6 Hz, 1H), 7.71 (dd, *J1* = 2 Hz, *J2* = 6.4 Hz, 1H), 7.53 (m, 4H), 7.33 (d, *J* = 8 Hz, 2H), 7.26 (d, *J* = 16 Hz, 1H), 6.93 (s, 1H), 2.63 (s, 2H), 2.52 (s, 2H), 2.36 (s, 2H), 1.02 (s, 6H); ^{13}C NMR (150 MHz, DMSO-d_6, 298 K) δ 170.71, 168.11, 155.53, 147.38, 140.96, 136.90.35.21, 135.03, 132.03, 130.77, 129.16, 128.49, 126.84, 124.92, 124.23, 122.83, 114.15, 113.34, 77.79, 42.71, 38.57, 32.14, 27.90, 21.30; HRMS: calculated to be [M-H]$^-$ 473.1085, found: 473.1092.

DMS-Br: 0.2 mmol, 60 mg, 57.8 %, ^{1}H NMR (400 MHz, DMSO-d_6, 298 K) δ 8.13 (d, *J* = 2 Hz, 1H), 7.74 (dd, *J1* = 2 Hz, *J2* = 8 Hz, 1H), 7.52 (m, 4H), 7.33 (d, *J* = 8 Hz, 2H), 7.26 (d, *J* = 16 Hz, 1H), 6.93 (s, 1H), 2.63 (s, 2H), 2.52 (s, 2H), 2.36 (s, 3H), 1.01 (s, 6H); ^{13}C NMR (150 MHz, DMSO-d_6, 298 K) δ 170.72, 168.08, 155.57, 148.74, 140.94,

137.11, 135.20, 134.98, 132.21, 131.98, 130.77, 129.04, 124.87, 124.20, 122.90, 116.50, 114.16, 113.35, 77.75, 42.71, 38.58, 32.14, 27.91, 21.31; HRMS: calculated to be $[M-H]^-$ 517.0580, found: 517.0596.

DMS-I: 0.2 mmol, 74 mg, 65.5 %, ^{1}H NMR (400 MHz, DMSO-d_6, 298 K) δ 8.27 (d, J = 2 Hz, 1H), 7.73 (dd, $J1$ = 2 Hz, $J2$ = 8 Hz, 1H), 7.52 (m, 4H), 7.54 (d, J = 8 Hz, 2H), 7.48 (d, J = 16 Hz, 1H), 7.38 (d, J = 8 Hz, 1H), 7.33 (d, J = 8 Hz, 2H), 6.92 (s, 1H), 2.61 (s, 2H), 2.51 (s, 2H), 2.36 (s, 3H), 1.01 (s, 6H); ^{13}C NMR (150 MHz, DMSO-d_6, 298 K) δ 170.75, 168.09, 155.72, 152.13, 140.88, 138.34, 137.00, 135.17, 135.06, 131.64, 130.75, 129.62, 124.04, 123.90, 92.36, 77.59, 42.73, 38.58, 32.13, 27.91, 21.31; HRMS: calculated to be $[M-H]^-$ 565.0441, found: 565.0462.

3. Measurement of spectroscopic properties

3.1 Fluorescence measurements in DMPC vesicles

3.1.1 Time responses of DMS-X to Sphinganine (Sph)

Solutions of 5 mM 1,2-dimyristoyl-sn-glycero-3-phosphocholine (DMPC) vesicles with or without Sph (200 μM) were treated with compounds **DMS-X** at a final concentration of 5 μM. Reactions were incubated on a tube rotator at 37 ºC for various time, respectively. Fluorescence emission spectra from 560-900 nm (λ_{ex} = 540 nm) for each sample were obtained on a Shimadzu RF-6000 spectrofluorophotometer.

3.1.2 Fluorescence responses of DMS-2F to Sph under different pH buffers

The pH control experiments were carried out in air at room temperature using a Sartorius PB-10 acidometer. The pH was adjusted by addition of HCl solution (1 M) and NaOH solution (1 M) to the PBS buffered system. Solutions of 5 mM DMPC vesicles under different pH buffer (from pH 3 to 9) with Sph (200 μM) were treated with **DMS-2F** at a final concentration of 5 μM. Reactions were incubated on a tube rotator at 37 ºC for 30 min.

3.1.3 Fluorescence spectra of DMS-2F within different analytes

To measure the response capacity of **DMS-2F** to different analyte, **DMS-2F** (5 μM) were incubated with various analyte, including ceramide (Cer), sphingomyelin (SM), glutathione (GSH), amino acids (Cys, D-Lys, L-Lys, Ser, Thr, Tyr), neurotransmitter: NE (norepinephrine), DA (dopamine) and EP (epinephrine) in DMPC vesicles at 37 °C for 30 min.

3.1.4 Fluorescent spectra of DMS-2F with different concentrations of Sph and the limit detection of DMS-2F to sph

To measure the fluorescence response of **DMS-2F** to different concentration of sph, **DMS-2F** (5 μM) were incubated with DMPC vesicles in PBS buffer (5 mM, pH = 7.4) containing different concentration of Sph (0 μM, 10 μM, 20 μM, 30 μM, 40 μM, 50 μM, 60 μM, 70 μM, 80 μM, 90 μM, 100 μM, 110 μM, 120 μM, 130 μM, 140 μM, 150 μM, 160 μM, 170 μM, 180 μM, 190 μM, 200 μM) for 30 min at 37 ºC.

The emission spectrum of free **DMS-2F** with DMPC vesicles in PBS buffer was collected for 20 times to confirm the background noise σ. The linear regression curve was then fitted according to the data in the range of sph from

0 to 110 μM. and obtained the slope of the curve, then the detection limit was calculated using the following equation:

$$\text{The limit of detection (LOD)} = 3\sigma/k$$

Where σ is the standard deviation of eleven blank measurements, and k is the slope of the linear equation. The detection limit (3σ/k) was then determined to be 9.33 ± 0.41 nM.

4. Cell culture

Rat adrenal pheochromocytoma cells (PC12), human lung cancer cells (A549), human glioma cells (U87), human embryonic lung fibroblast cells (MRC-5) and human hepatocellular carcinomas cells (Hep G2) were obtained from the American Type Culture Collection (Manassas, VA). Cells were cultured in Dulbecco's Modified Eagle's medium (DMEM, PAN Biotech, Germany) supplemented with 10% fetal bovine serum (Life Technologies, USA), sodium pyruvate, non-essential amino acids and 100 U/mL penicillin/100 U/mL streptomycin, and grown at 37 ºC in a 5% CO_2 incubator.

5. MTT assay

3-(4,5-dimethylthiazol-2-yl)-2,5-diphenyltetrazolium bromide (MTT) assays were carried out to evaluate the toxicity of probe **DMS-X**. Rat adrenal pheochromocytoma cells (PC12), human lung cancer cells (A549), human glioma cells (U87), human embryonic lung fibroblast cells (MRC-5) and human hepatocellular carcinomas cells (HepG2) were seeded into 96-well microtiter plates with total volumes of 100 μL well^{-1}. After 12 h of incubation, various concentrations of **DMS-X** (0 μM, 100 μM, 50 μM, 25 μM, 12.5 μM, 6.25 μM, 3.125 μM, and 1.56 μM) were added, and the cells were cultured for another 48 h. Subsequently, an MTT solution (50 μL, 5 mg mL-1, in DMEM) was added to each well. After 4 h, the MTT solution was removed, and DMSO (150 μL) was added to each well. Finally, the absorbance at 570 nm was measured using a Triturus microplate reader.

6. Fluorescent imaging studies

6.1 Time-response of DMS-2F to Sph in living cells

Living cells were plated in a confocal dish at a density of 80,000 cells/well and allowed to attach overnight. Media was removed and each well washed with DMEM (containing 10% FBS) and then 1000 μL media containing 10 μM **DMS-2F** was added and cells were incubated at 37 ºC, 5 % CO_2 for 30 min. Media in each well was then exchanged for 1000 μL of media containing 20 μM Sph and the cells were incubated at 37 ºC, 5 % CO_2 for different time before imaging. Fluorescence image was obtained with a fluorescence microscope (Axio Observer Z1 ZEISS) from the fluorescence microscope software Apotome2.

6.2 Dose-response of DMS-2F to Sph in living cells

Living cells were plated in a confocal dish at a density of 80,000 cells/well and allowed to attach overnight. Media was removed and each well washed with DMEM (containing 10% FBS) and then 1000 μL media containing 10 μM **DMS-2F** was added and cells were incubated at 37 ºC, 5 % CO_2 for 30 min. Media in each well was then exchanged for 1000 μL of media containing the various concentrations of Sph and the cells were incubated at 37 ºC, 5 % CO_2 for 2 h before imaging.

6.3 Detection of endogenous Sph in living cells

Living cells were plated in a confocal dish at a density of 80,000 cells/well and allowed to attach overnight. Media was removed and each well washed once with Opti-MEM media (FBS-free) before adding 1000 μL Opti-MEM (FBS-free) containing 10 μM **DMS-2F**. Cells were incubated at 37 ºC, 5 % CO_2 for 2 h. Media was removed and each well washed three times with PBS before imaging.

6.4 Detection of endogenous Sph variations induced by $A\beta_{42}$ monomer and $A\beta_{42}$ oligomers in PC12 cells

PC12 cells were plated in a confocal dish at a density of 80,000 cells/well and allowed to attach 12 h. Media in each well was then exchanged for 1000 μL of media containing the various concentrations of $A\beta_{42}$ monomer (0, 2.5, 5 and 10 μM) and $A\beta_{42}$ oligomers (0, 2.5, 5 and 10 μM) and the cells were incubated at 37 ºC, 5 % CO_2 for 12 h. And then, media was removed and each well washed with DMEM (containing 10% FBS) and then 1000 μL media containing 10 μM **DMS-2F** and incubated at 37 ºC, 5 % CO_2 for 2 h and rinsed with PBS before imaging.

6.5 Detection of endogenous Sph variations induced by H_2O_2 in PC12 cells

PC12 cells were plated in a confocal dish at a density of 80,000 cells/well and allowed to attach 12 h. Media in each well was then exchanged for 1000 μL of media containing the various concentrations of H_2O_2 (0, 50, 100 and 200 μM) and the cells were incubated at 37 ºC, 5 % CO_2 for 4 h. And then, media was removed and each well washed with DMEM (containing 10% FBS) and then 1000 μL media containing 10 μM **DMS-2F** and incubated at 37 ºC, 5 % CO_2 for 2 h and rinsed with PBS before imaging.

6.6 Detection of endogenous Sph variations induced by $A\beta_{42}$ oligomers and H_2O_2 in different cell lines

Different cell lines (PC12, U87, HepG2, A549, and MRC5) were plated in the confocal dish at a density of 40,000 cells/well and allowed to attach 12 h. Media in each well was then exchanged for 500 μL of media containing 10 μM $A\beta_{42}$ oligomers and 100 μM H_2O_2, respectively, the cells were incubated at 37 ºC, 5 % CO_2 for 12 h and 6 h, respectively. Then, the media was removed, and each well washed with DMEM (containing 10% FBS) and then 500 μL media containing 10 μM **DMS-2F** was added and incubated at 37 ºC, 5 % CO_2 for 2 h and rinsed with PBS before imaging.

6.7 Detection of Sph variations induced by Aβ$_{42}$ oligomers and H_2O_2 under the preincubated of different inhibitors in PC12cells

PC12 cells were plated in a confocal dish at a density of 40,000 cells/well and allowed to attach 12 h. Media in each well was then exchanged for 500 µL of media containing indicated concentrations of inhibitors (10 µM AM, 20 µM GW4869, 10 µM LCL-52, 500 µM NAC, and 1mM Trolox) for 2h, and then treated with 10 µM Aβ$_{42}$ oligomers and 50 µM H_2O_2 at 37 ºC, 5 % CO_2 for 12 h and 4 h, respectively. Then, the media was removed and each well was washed with DMEM (containing 10% FBS) and then 500 µL media containing 10 µM **DMS-2F** and incubated at 37 ºC, 5 % CO_2 for 2 h and rinsed with PBS before imaging.

7. ROS detection.

The effect of RPNSs on intracellular ROS generation in cancer cells was measured with a Reactive Oxygen Species Assay Kit (Beyotime) following the manufacturer's protocol. Briefly, PC12 cells were seeded in the confocal dish at a density of 40,000 cells/well for 12 h and then treated with 1 µM Aβ$_{42}$ oligomers for different times (0 h, 0.5 h, 1.0 h, 2.0 h, 4.0 h, 8.0 h, and 10.0 h). After treatment, the medium was removed, and the cells were washed 3 times with serum-free DMEM. DCF-DA/serum-free medium (final concentration is 10 µM) was added to the well and incubated with cells for 30 min at 37°C. The fluorescence image of DCF was obtained with a fluorescence microscope (SpinSR10).

8. Western blot analysis.

PC12 cells were cultured in 6-cm culture dishes and allowed to attach for 12 h. Then, the cells were treated with 1 µM Aβ$_{42}$ oligomers for 0 h, 2 h, 4 h, 6.0 h, 8.0 h, and 10.0 h, respectively and 50 µM H_2O_2 for different times (0 h, 1.0 h, 2.0 h, 3.0 h, 4.0 h, and 5.0 h), respectively, and the total protein was obtained using a Whole Cell Lysis Assay Kit (KeyGEN), and the protein concentration was determined with an Enhanced BCA Protein Assay Kit (Beyotime). Protein samples were separated by 10% SDS-PAGE and transferred to PVDF membranes. The membrane was first blocked with 5% nonfat dry milk in TBST at room temperature for 1 h and then incubated with antibodies at 4° C overnight. The expression of *β*-actin was used as the internal standard. All antibodies were purchased from ABclonal.

9. Immunocytochemistry.

PC12 cells were first seeded at a density of 40,000 cells/well and treated with Aβ$_{42}$ oligomers (1 µM) and H_2O_2 (50 µM) for 8 h and 3 h, respectively. After immobilization with 4% paraformaldehyde for 15 min, the cells were washed three times with PBS and permeabilized with PBS containing 0.2% Triton X-100. The cells were blocked with FBS (55%) in PBST for 30 min and then incubated with antibodies at 4°C overnight. Finally, the cells were monitored using a fluorescence microscope (Olympus, SpinSR10). The related antibodies were obtained from ABclonal.

10. Live Imaging of Zebrafish.

Zebrafish were maintained in E3 embryo media (5 mM NaCl, 0.15 mM KCl, 0.33 mM $MgSO_4$, and 0.33 mM $CaCl_2$, pH = 7.0 ± 1.0) for 3 days, then placed in glass-bottom dishes and preincubated with inhibitors AMI, GW4869, LCL-521, and Trolox for 2 h, the media was exchanged for media containing 10 μM $A\beta_{42}$ oligomers for 12 h at 28 °C. Then, the media was further exchanged 1000 μL media containing 10 μM **DMS-2F** and incubated at 28 ºC for 2 h and rinsed with PBS before imaging. Finally, the treated zebrafish were imaged with an inverted fluorescence microscope (Olympus, SpinSR10).

Quantification of cellular fluorescence

Using Image J, the mean fluorescence of individual cells was measured within 3 images taken across at least 2 biological replicates for each condition. The mean cellular fluorescence intensity was then calculated for each of the 3 images and data was reported as the mean of these 3 means ± SD.

Statistical method

All the dates are expressed as mean ± standard deviation. Differences between different experimental groups were analyzed by two-tailed Student's test. One-way analysis of variance (ANOVA) was used in multiple group comparisons. Difference with $^{*}P \le 0.05$, $^{**}P \le 0.01$, $^{***}P \le 0.001$, $^{****}P \le 0.0001$ was considered statistically significant.

Results and Discussion

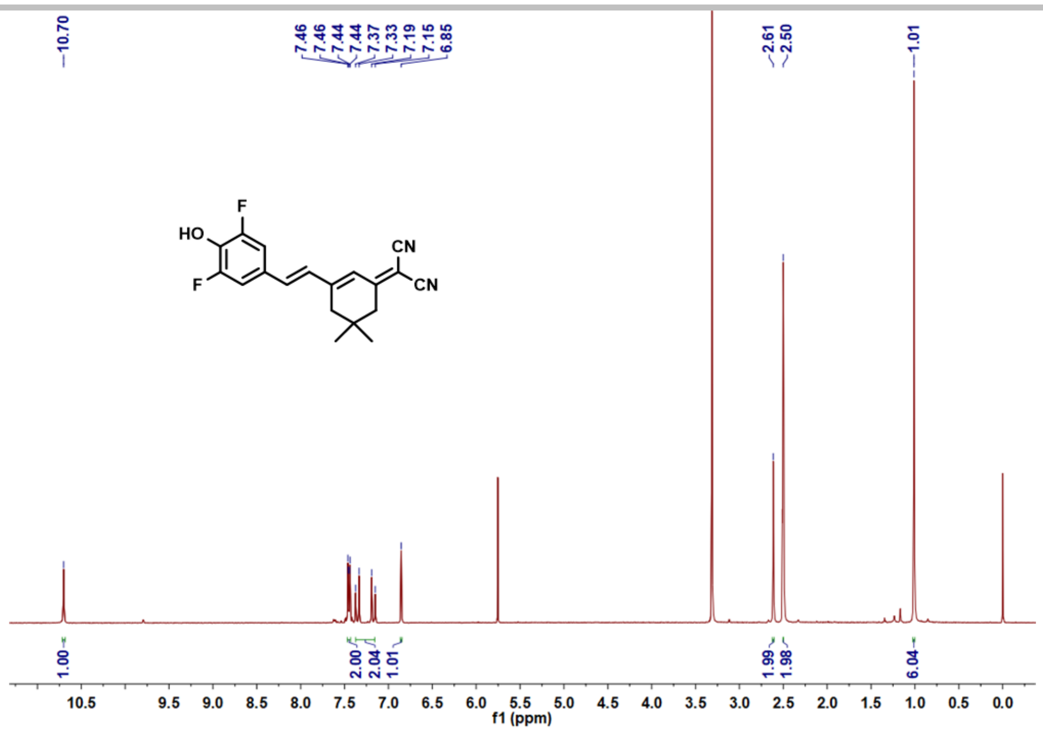

Figure S1: ^{1}H NMR spectrum of **DM-2F** (400 MHz, DMSO-d_6, 298 K)

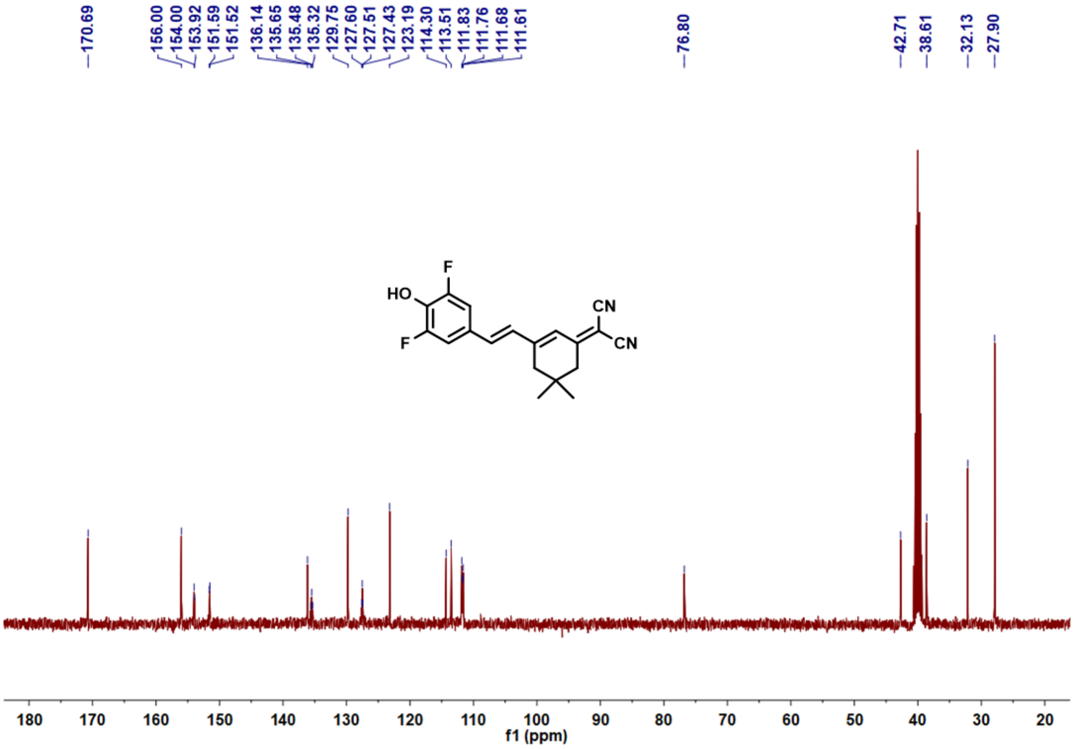

Figure S2: ^{13}C NMR spectrum of **DM-2F** (150 MHz, DMSO-d_6, 298 K)

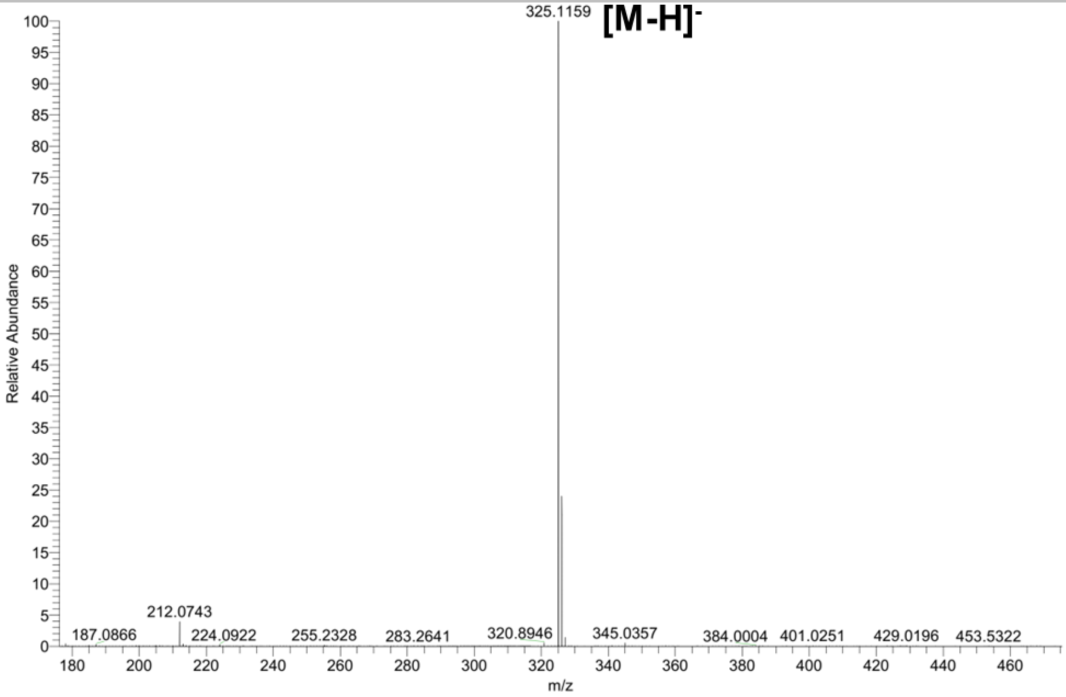

Figure S3: High resolution mass spectrum of **DM-2F** (MeOH, 298 K)

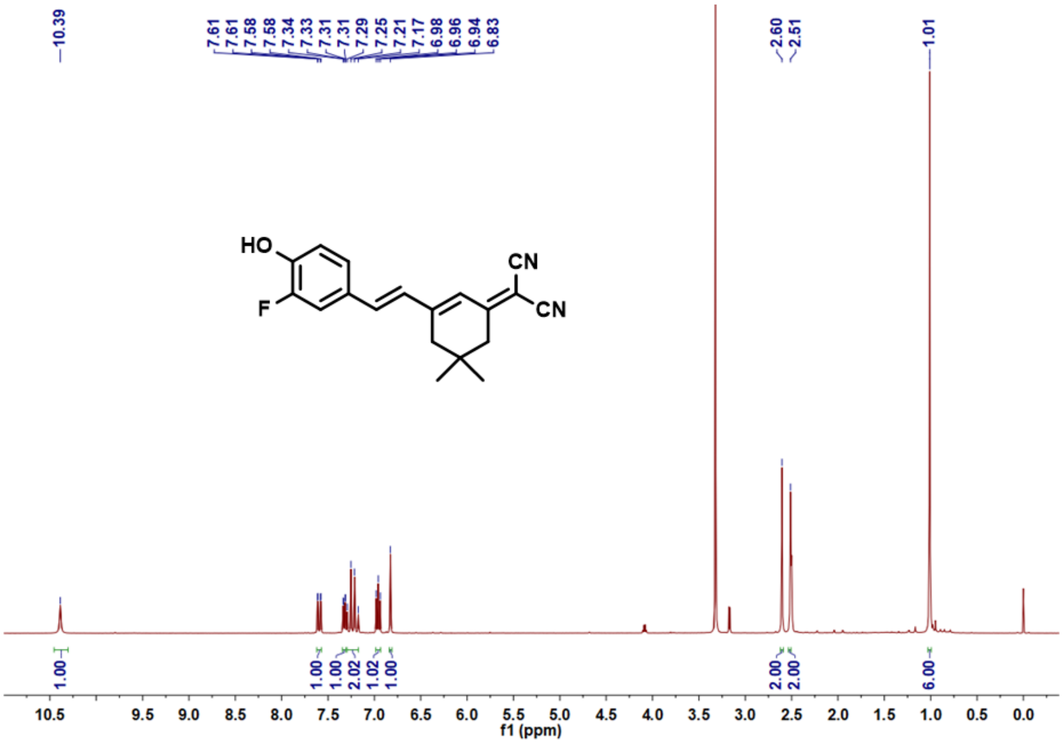

Figure S4: ^{1}H NMR spectrum of **DM-F** (400 MHz, DMSO-d_6, 298 K)

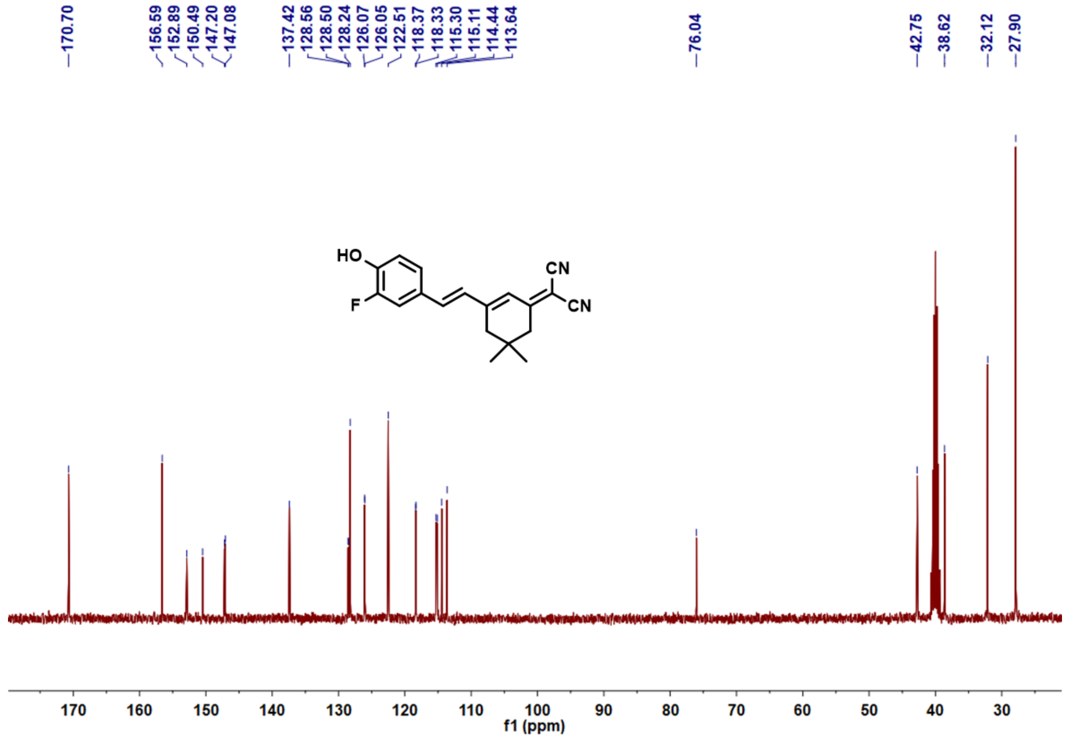

Figure S5: ^{13}C NMR spectrum of **DM-F** (150 MHz, DMSO-d_6, 298 K)

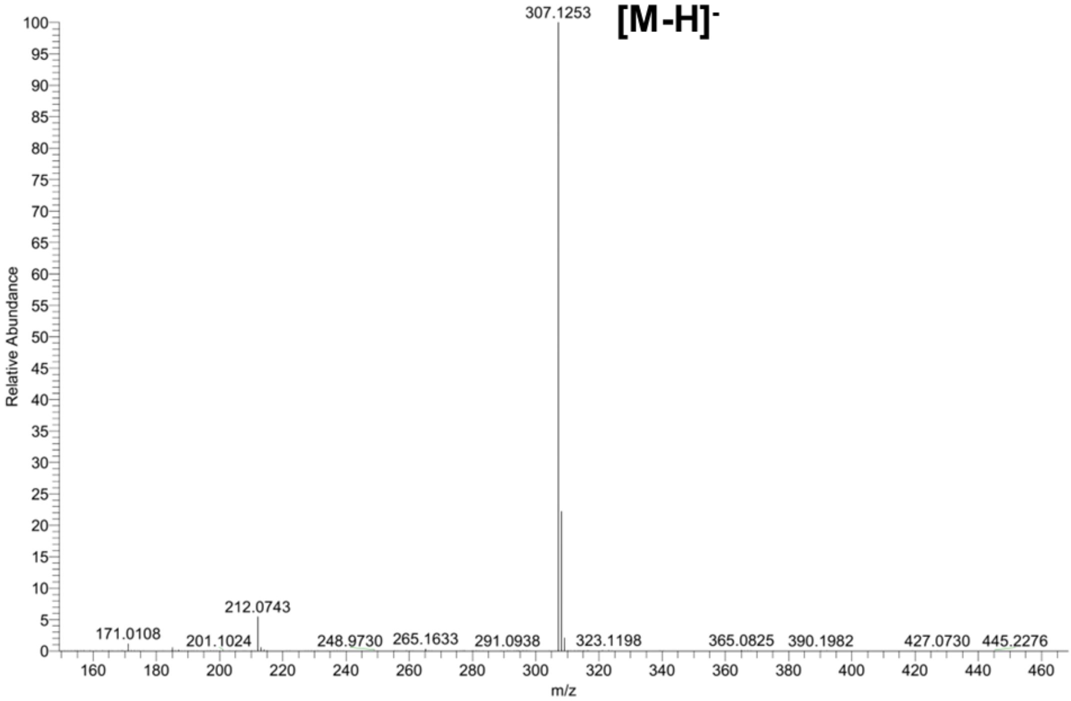

Figure S6: High resolution mass spectrum of **DM-F** (MeOH, 298 K)

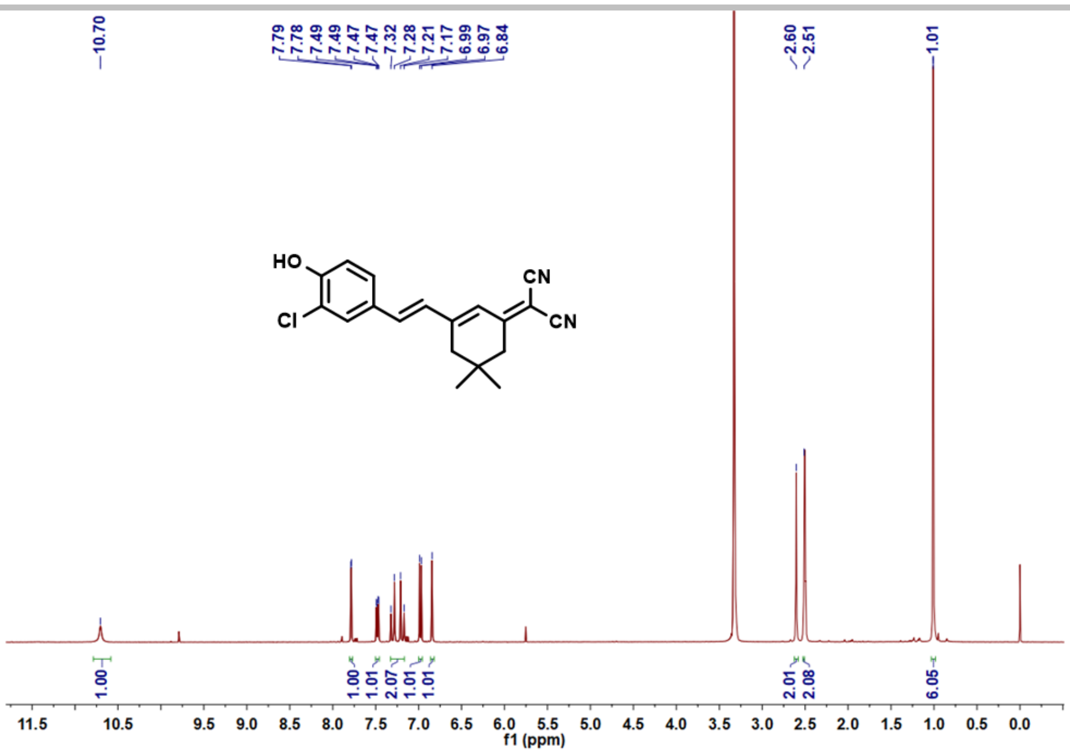

Figure S7: ^{1}H NMR spectrum of **DM-Cl** (400 MHz, DMSO-d_6, 298 K)

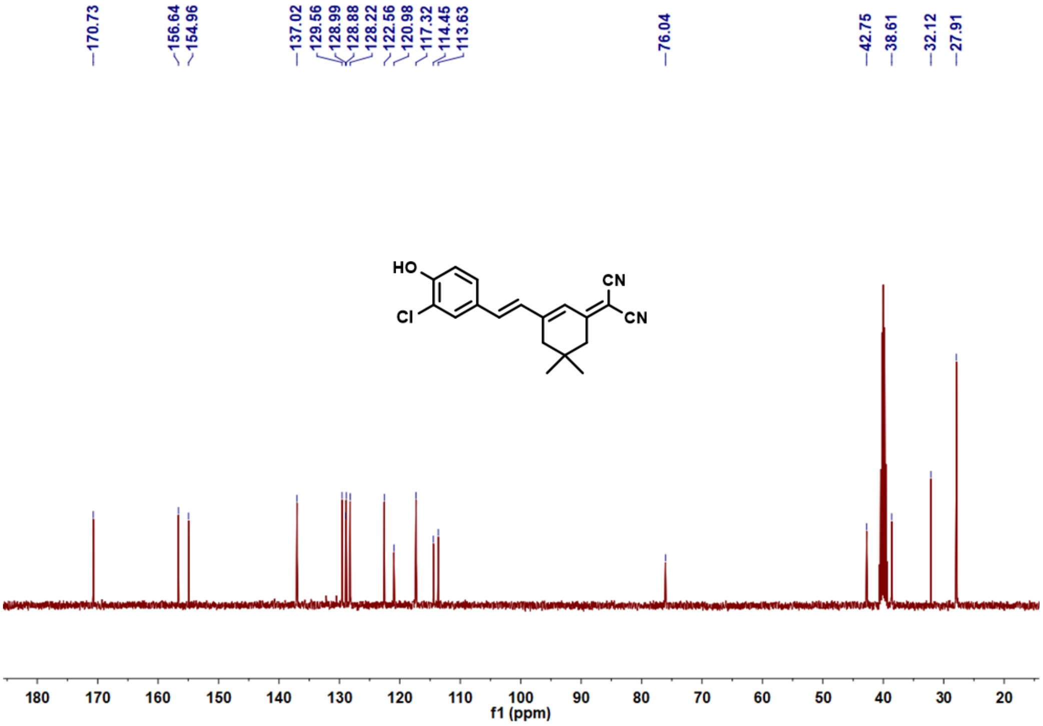

Figure S8: ^{13}C NMR spectrum of **DM-Cl** (150 MHz, DMSO-d_6, 298 K)

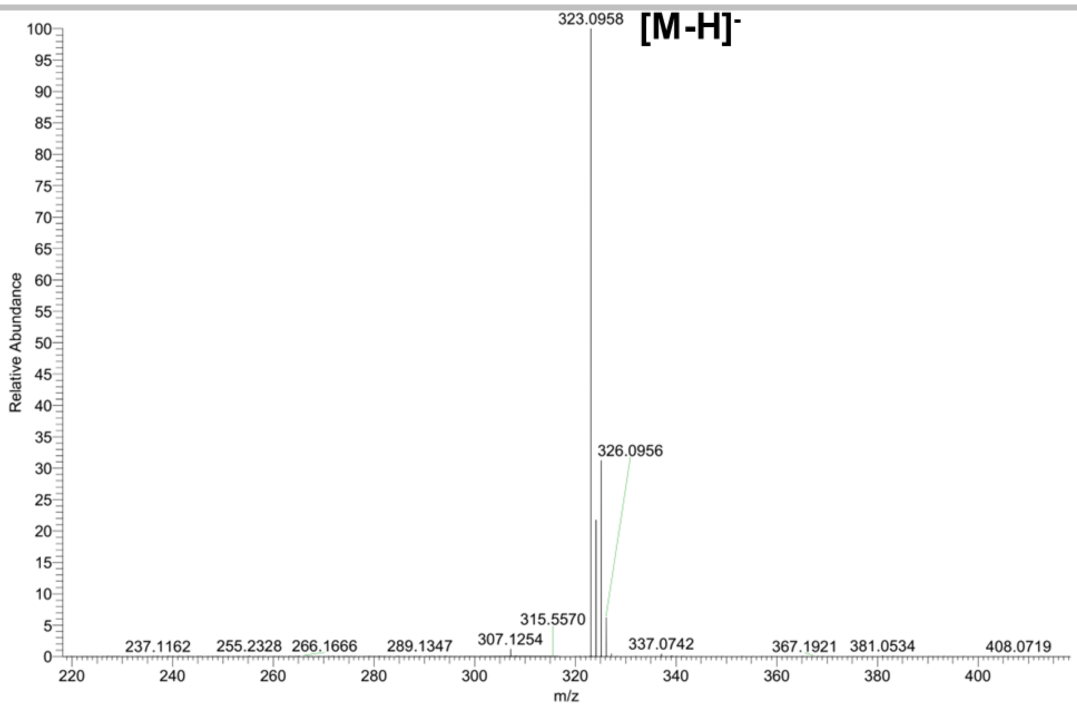

Figure S9: High resolution mass spectrum of **DM-Cl** (MeOH, 298 K)

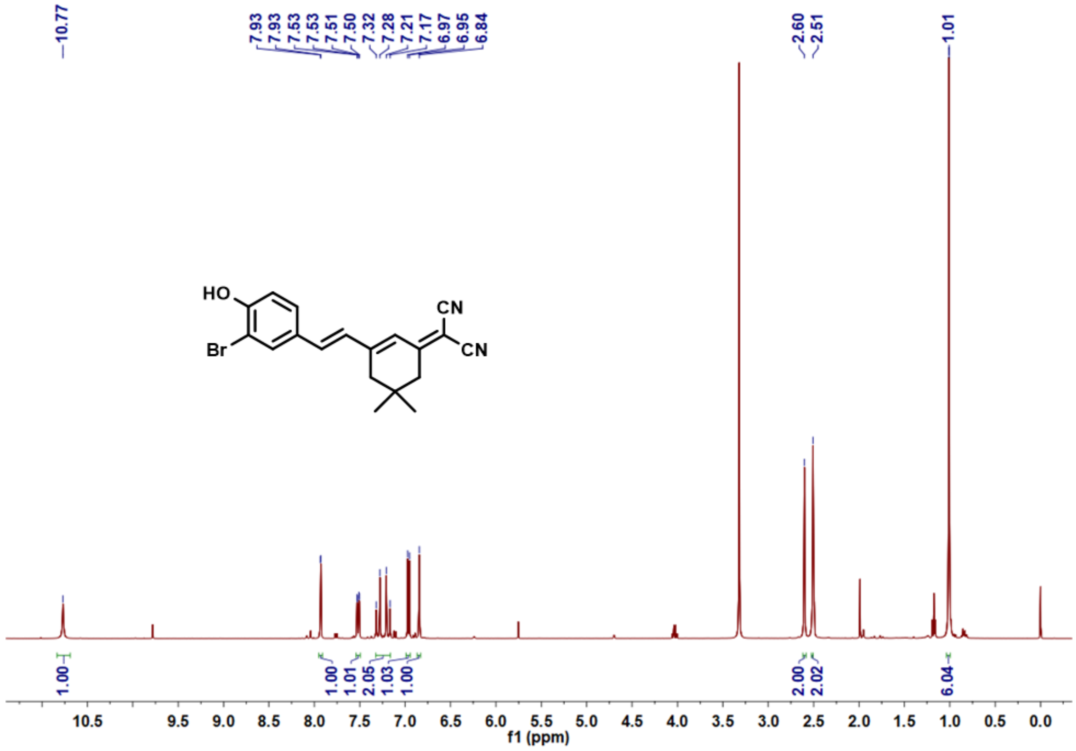

Figure S10: ^{1}H NMR spectrum of **DM-Br** (400 MHz, DMSO-d_6, 298 K)

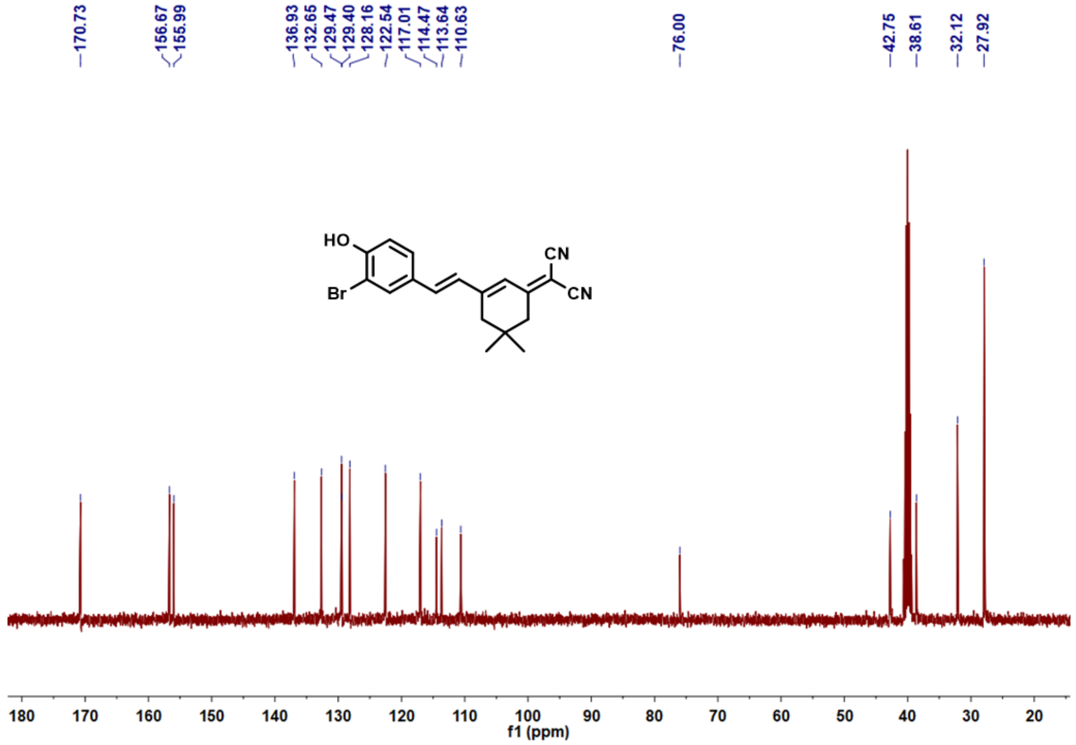

Figure S11: ^{13}C NMR spectrum of **DM-Br** (150 MHz, DMSO-d_6, 298 K)

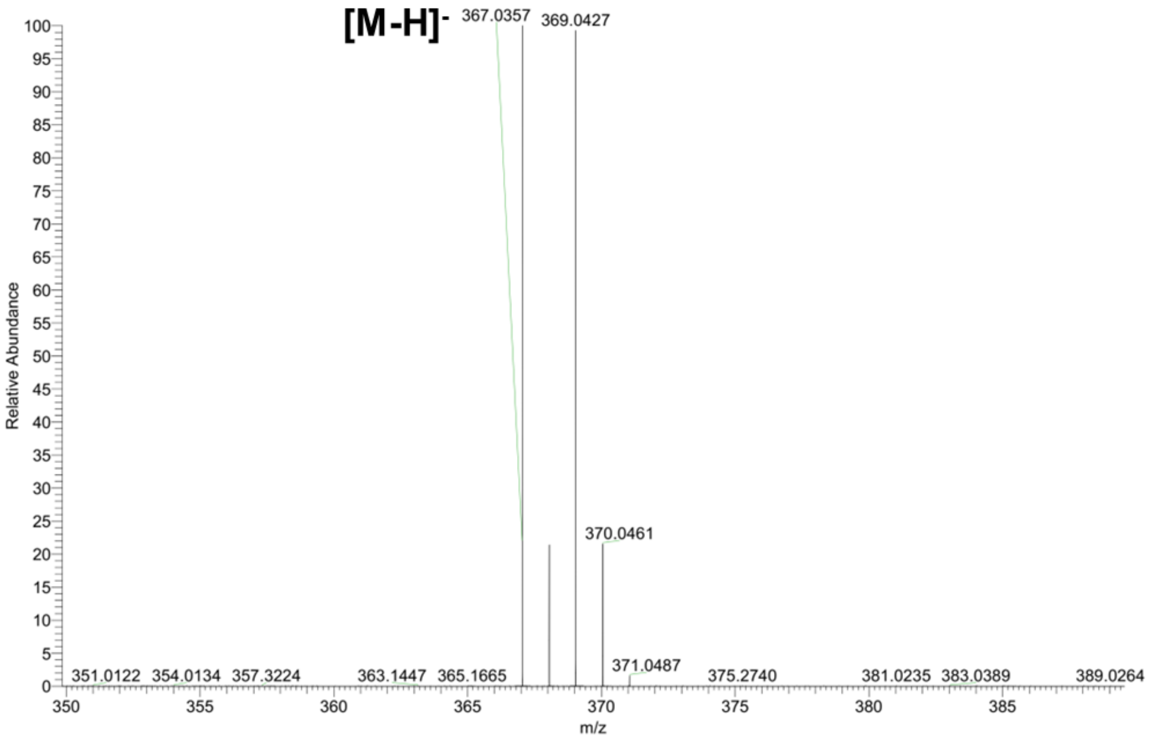

Figure S12: High resolution mass spectrum of **DM-Br** (MeOH, 298 K)

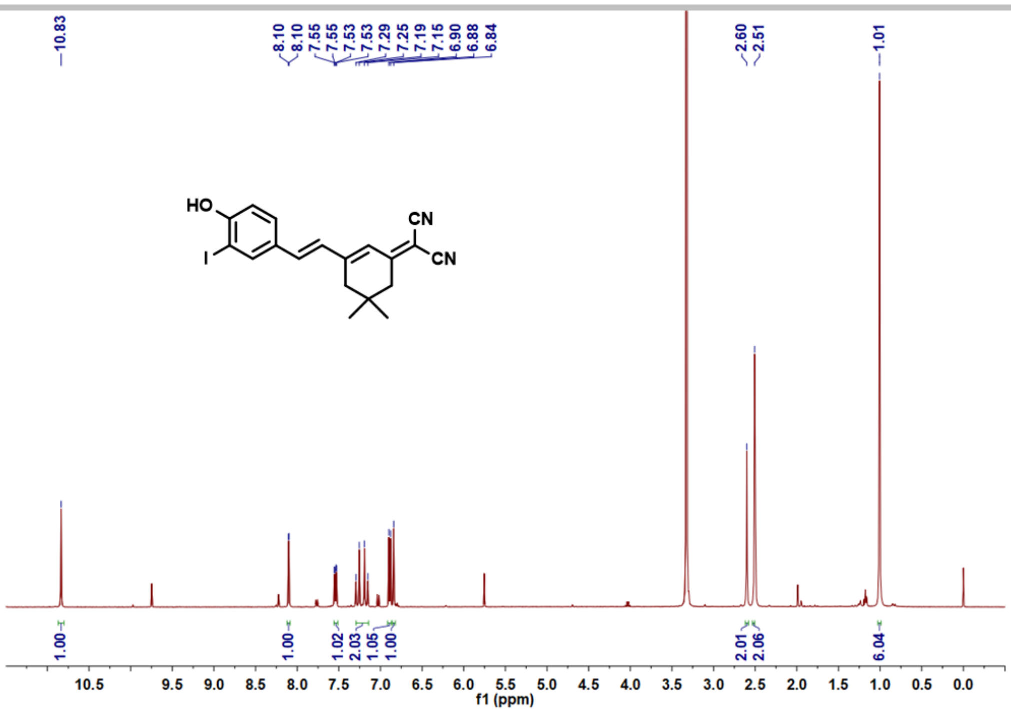

Figure S13: ^{1}H NMR spectrum of **DM-I** (400 MHz, DMSO-d_6, 298 K)

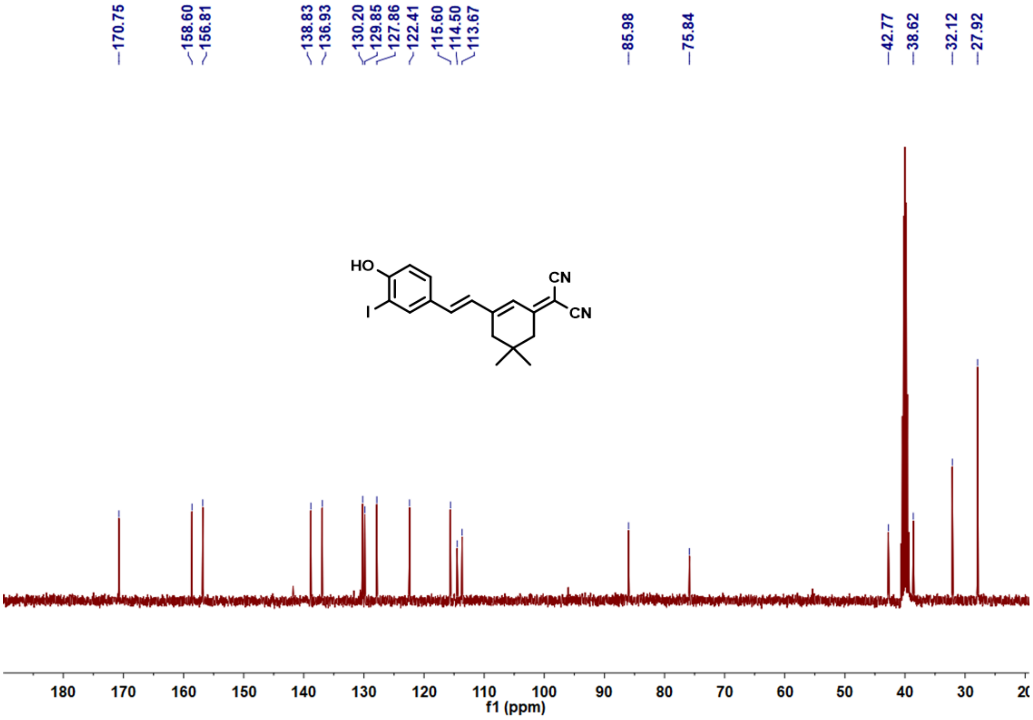

Figure S14: ^{13}C NMR spectrum of **DM-I** (150 MHz, DMSO-d_6, 298 K)

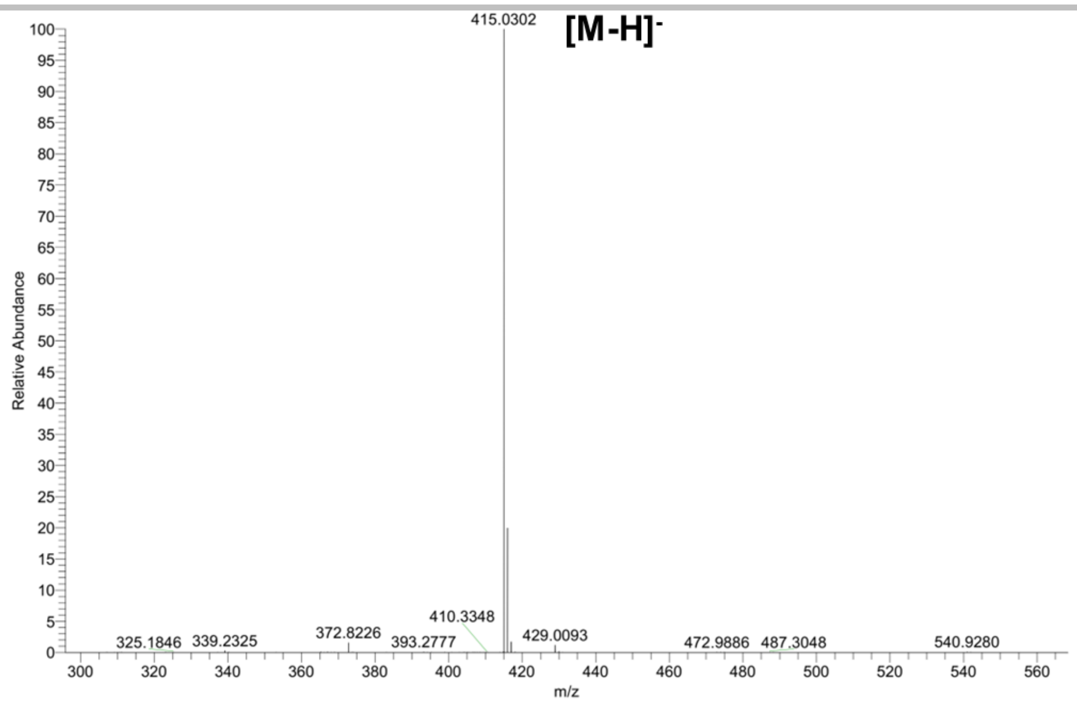

Figure S15: High resolution mass spectrum of **DM-I** (MeOH, 298 K)

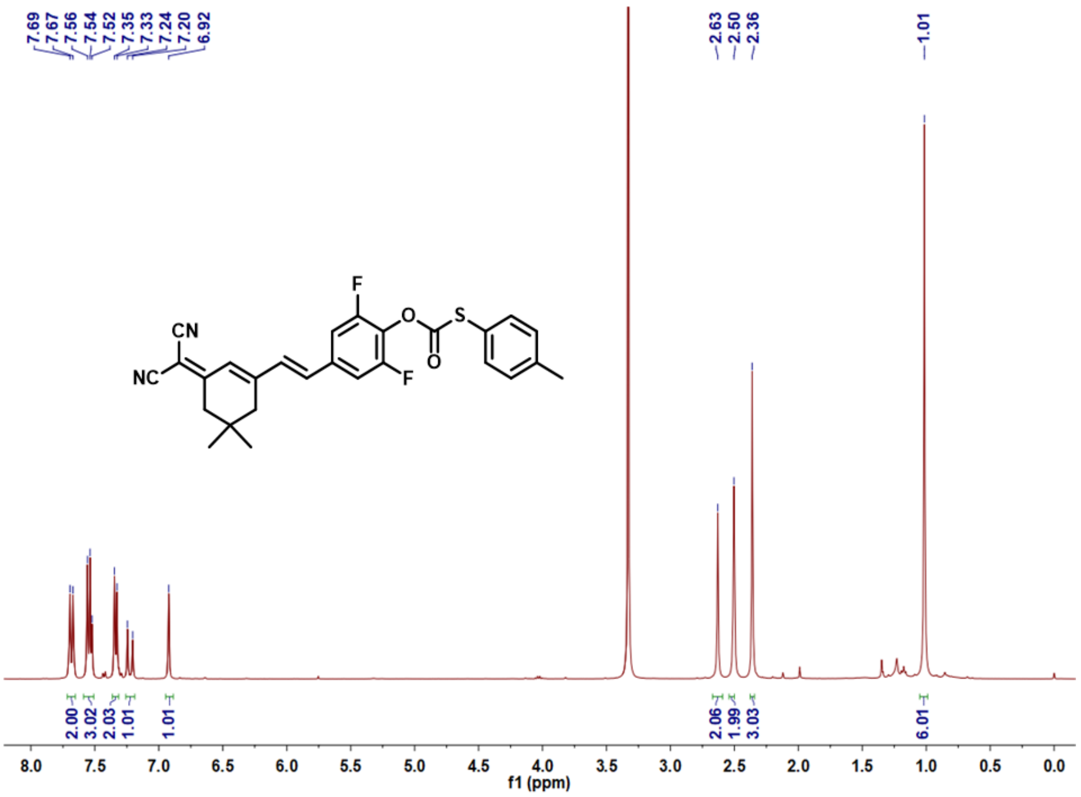

Figure S16: ^{1}H NMR spectrum of **DMS-2F** (400 MHz, DMSO-d_6, 298 K)

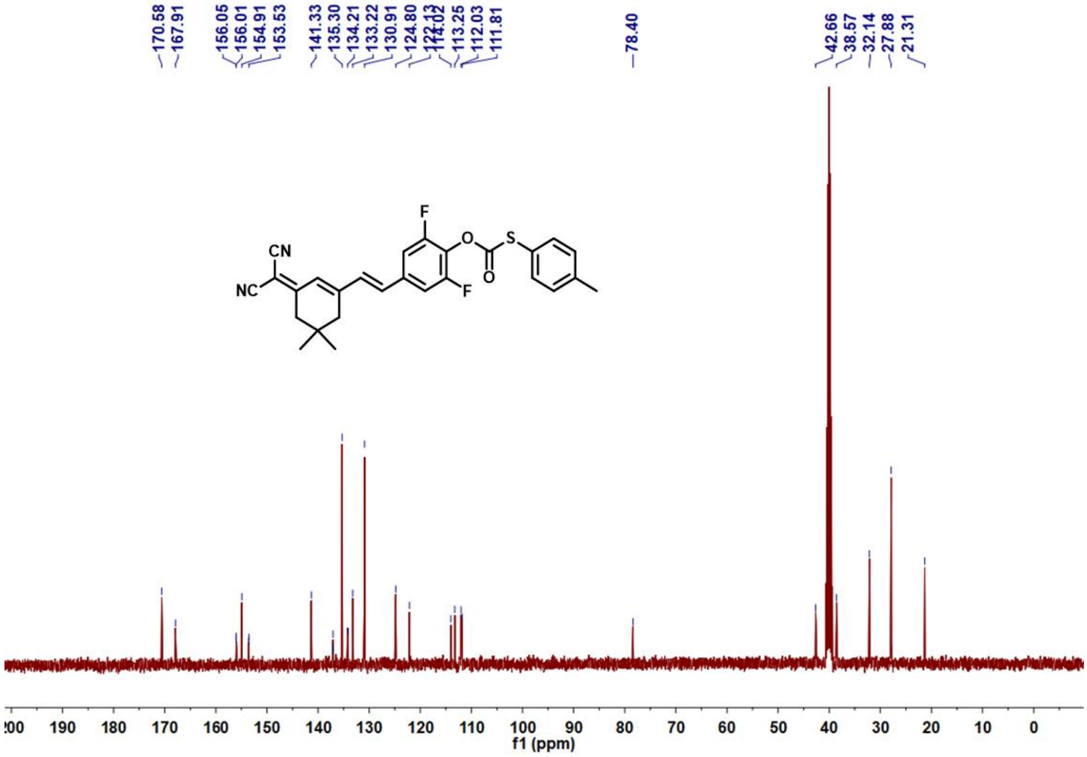

Figure S17: ^{13}C NMR spectrum of **DMS-2F** (150 MHz, DMSO-d_6, 298 K)

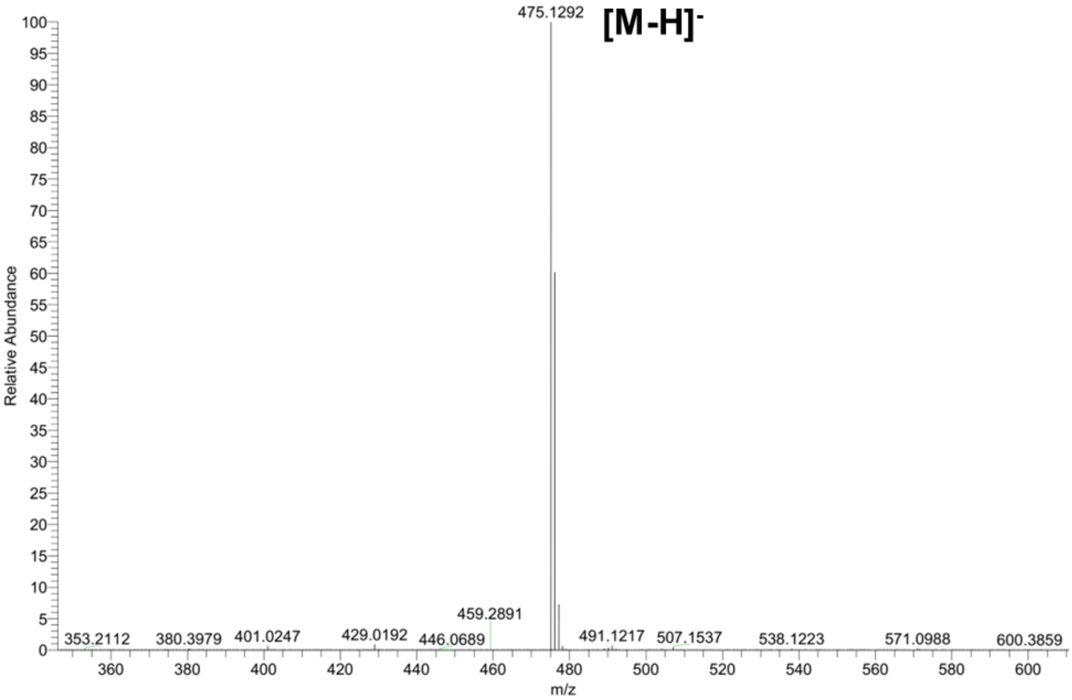

Figure S18: High resolution mass spectrum of **DMS-2F** (MeOH, 298 K)

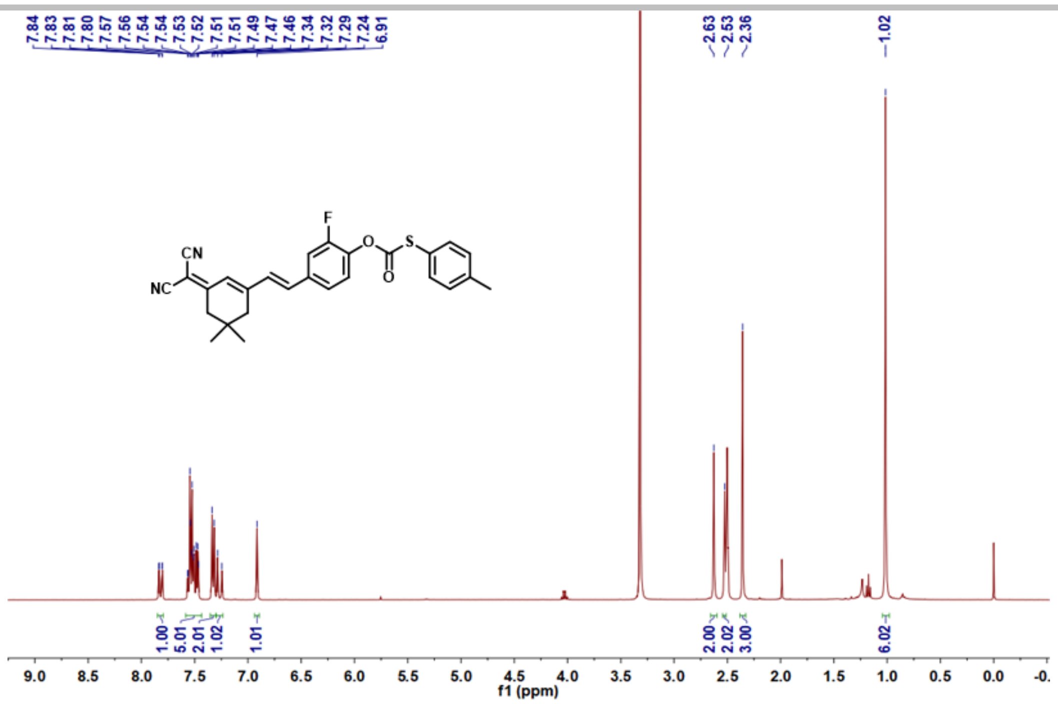

Figure S19: ^{1}H NMR spectrum of **DMS-F** (400 MHz, DMSO-d_6, 298 K)

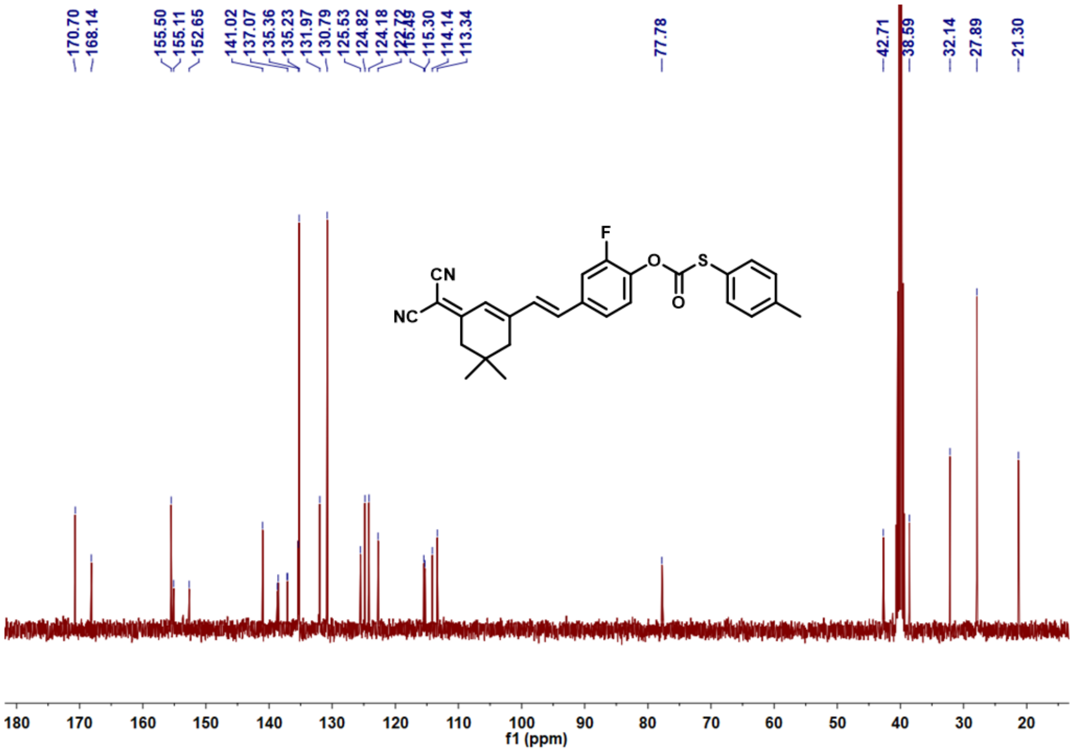

Figure S20: ^{13}C NMR spectrum of **DMS-F** (150 MHz, DMSO-d_6, 298 K)

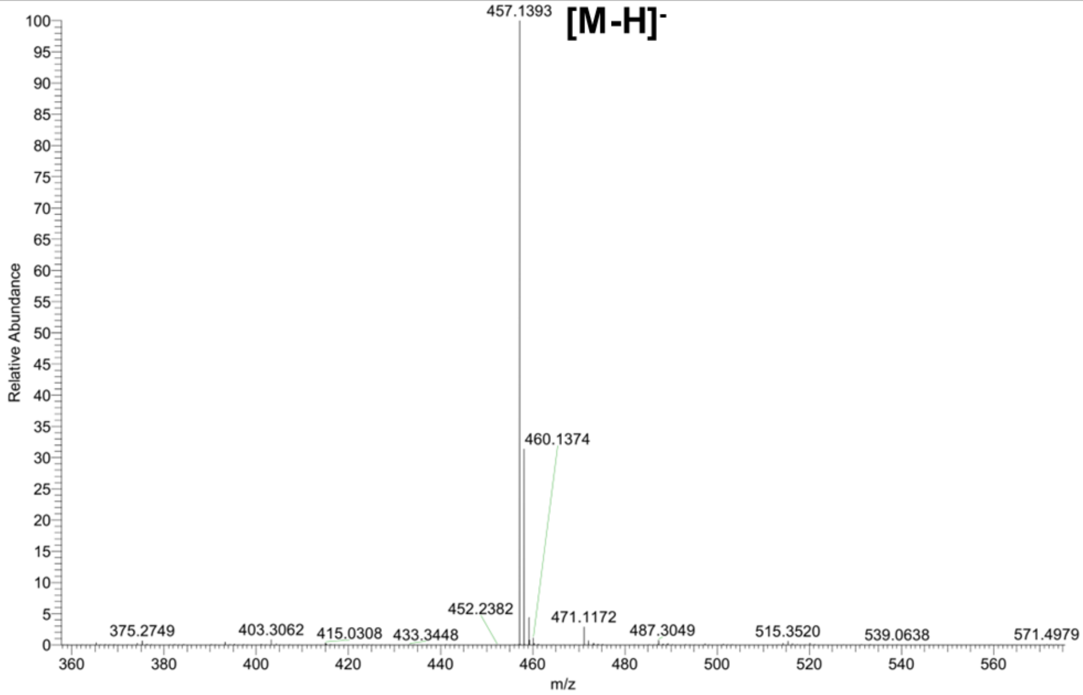

Figure S21: High resolution mass spectrum of **DMS-F** (MeOH, 298 K)

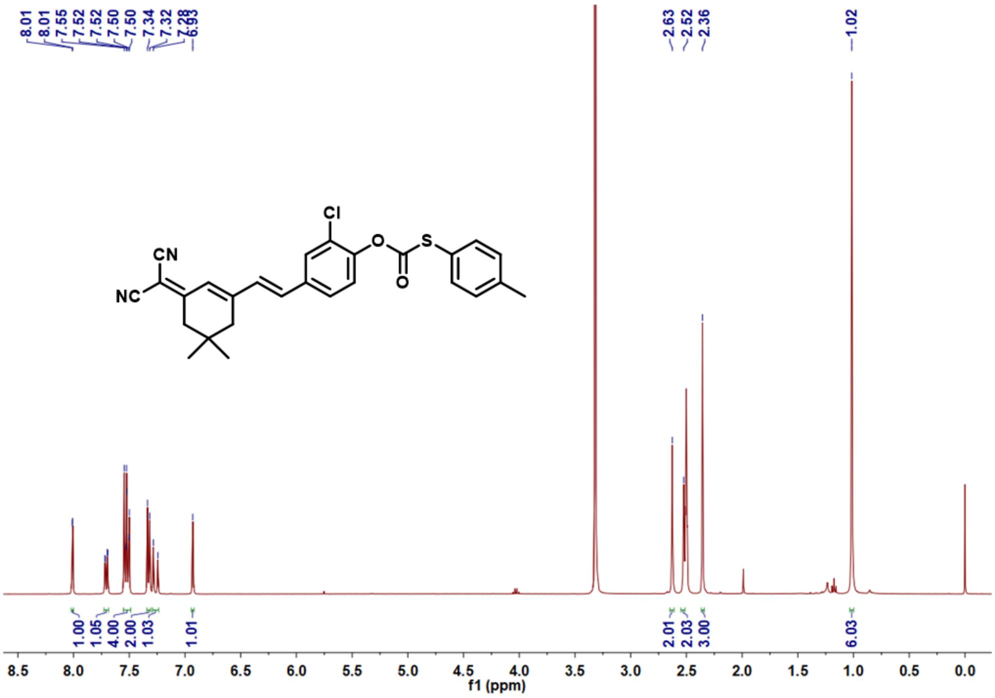

Figure S22: ^{1}H NMR spectrum of **DMS- Cl** (400 MHz, DMSO-d_6, 298 K)

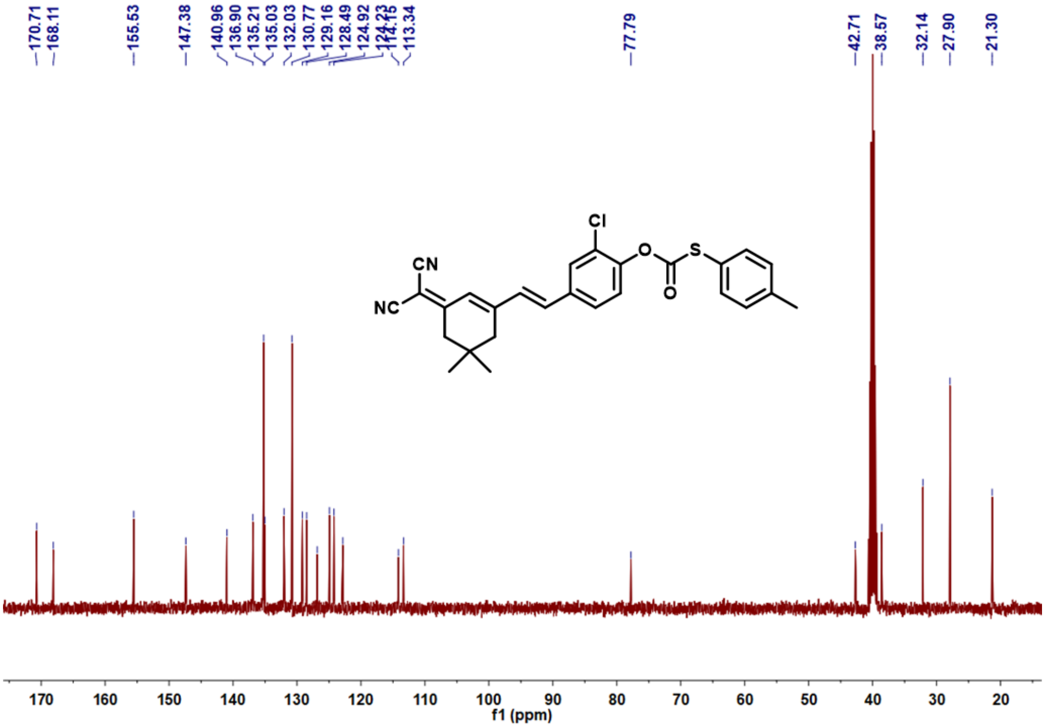

Figure S23: ^{13}C NMR spectrum of **DMS- Cl** (150 MHz, DMSO-d_6, 298 K)

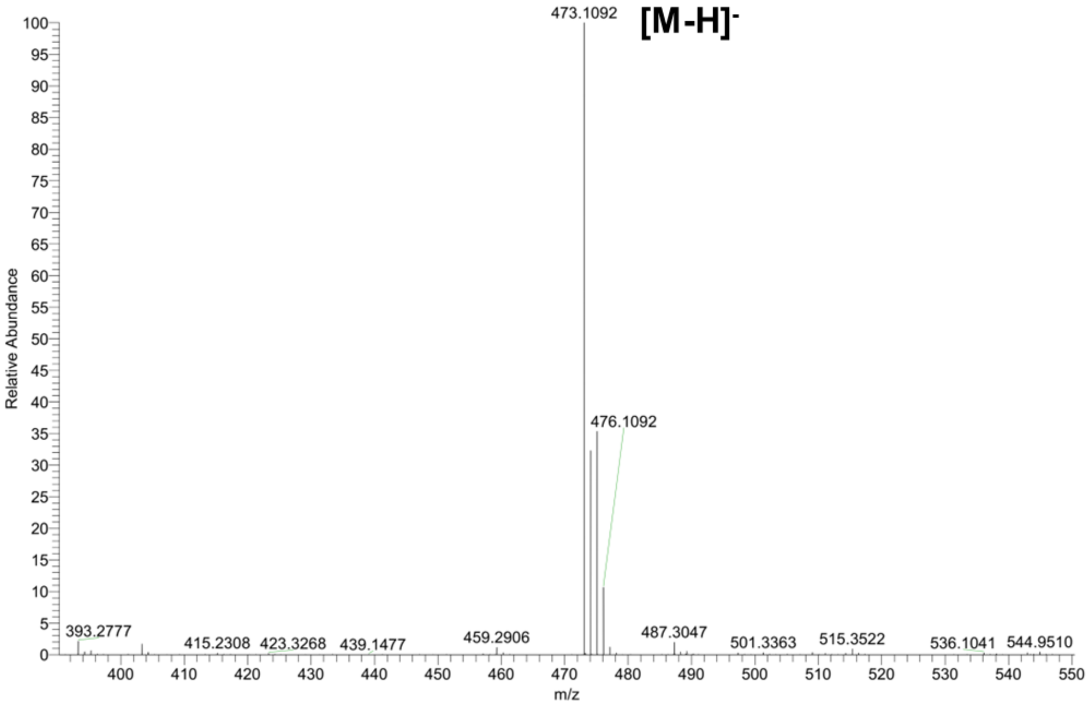

Figure S24: High resolution mass spectrum of **DMS-Cl** (MeOH, 298 K)

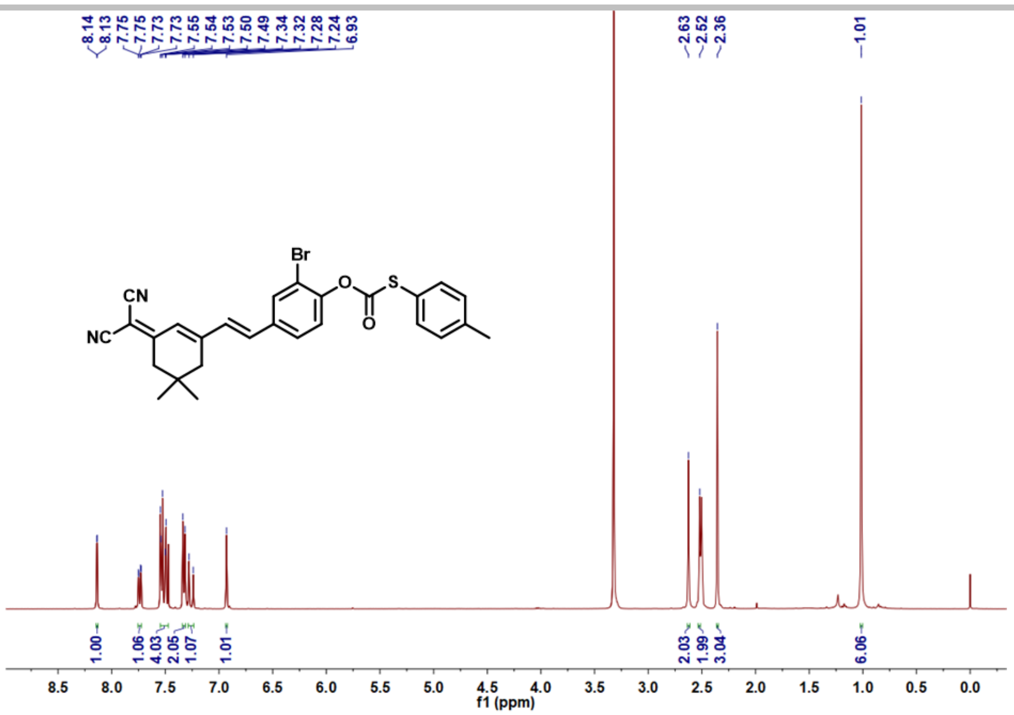

Figure S25: ^{1}H NMR spectrum of **DMS-Br** (400 MHz, DMSO-d_6, 298 K)

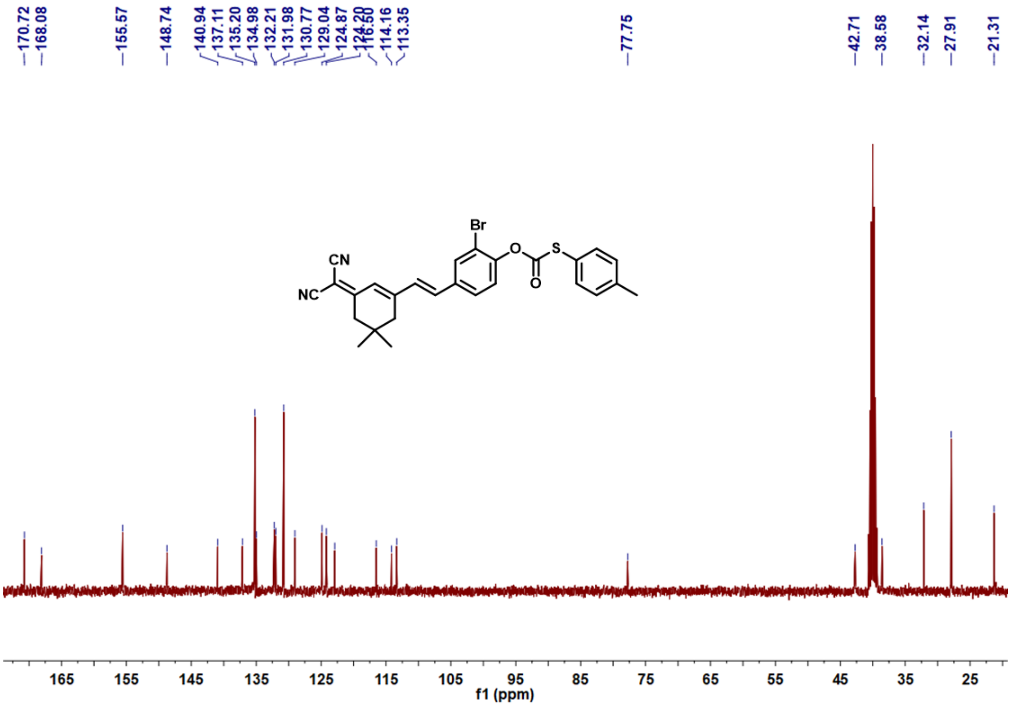

Figure S26: ^{13}C NMR spectrum of **DMS-Br** (150 MHz, DMSO-d_6, 298 K)

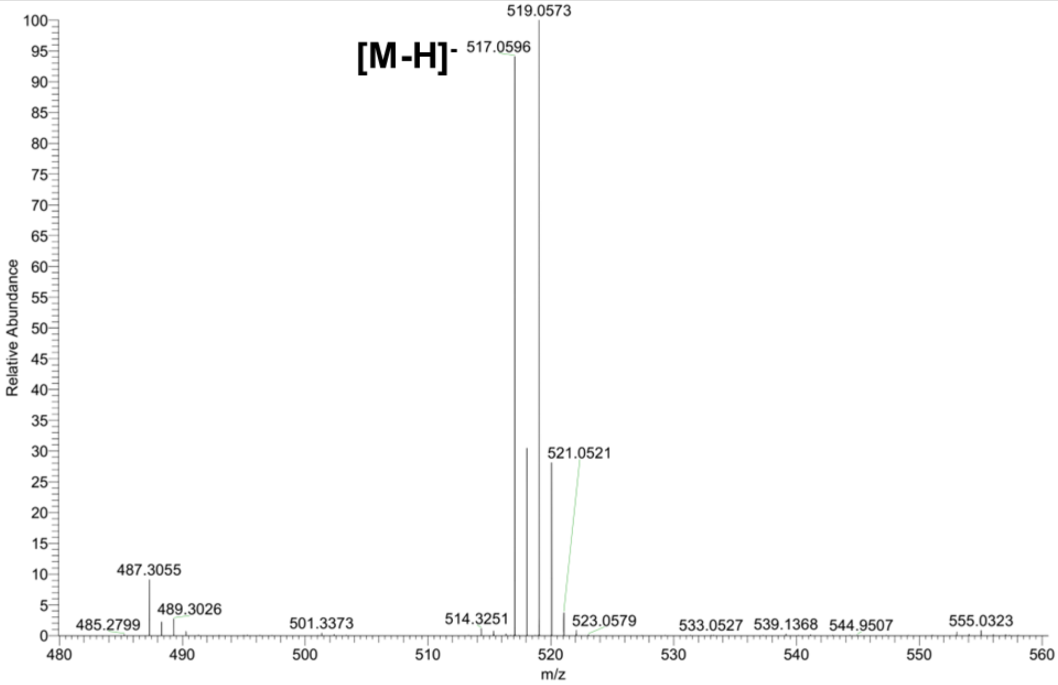

Figure S27: High resolution mass spectrum of **DMS-Br** (MeOH, 298 K)

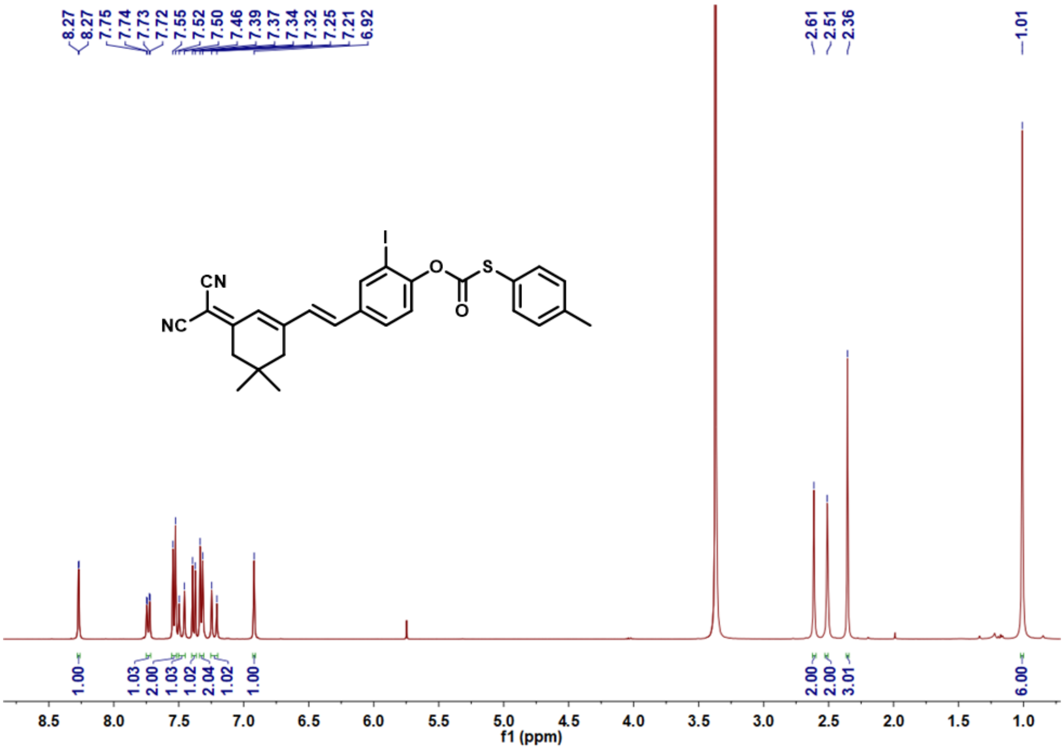

Figure S28: ^{1}H NMR spectrum of **DMS-I** (400 MHz, DMSO-d_6, 298 K)

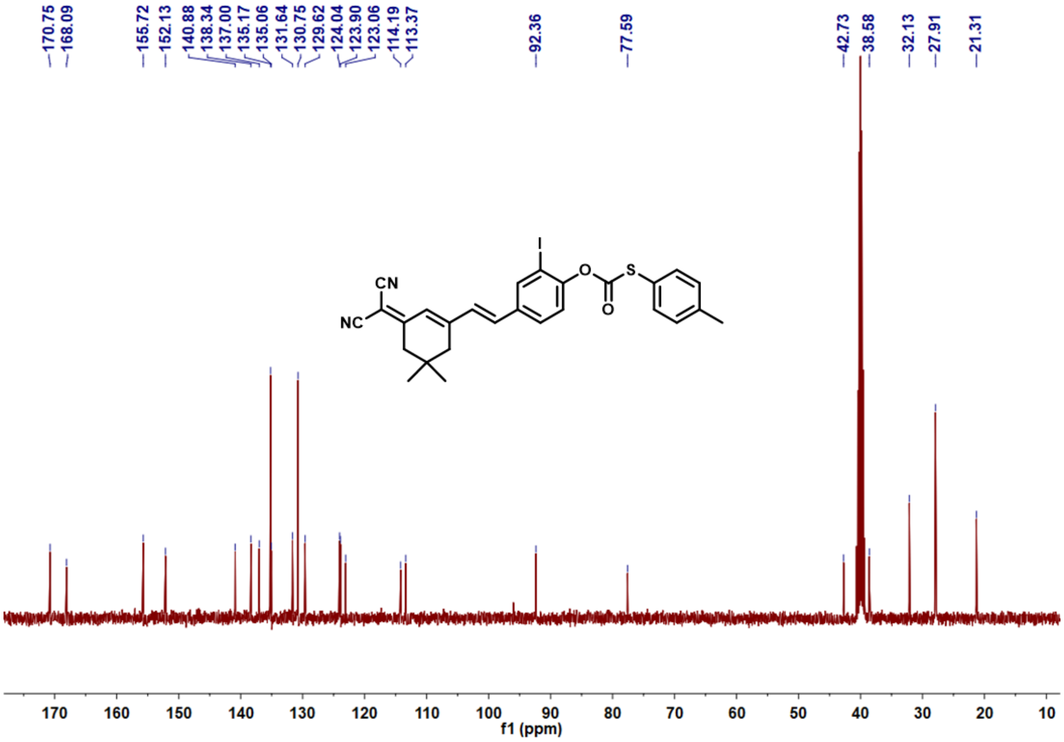

Figure S29: ^{13}C NMR spectrum of **DMS-I** (150 MHz, DMSO-d_6, 298 K)

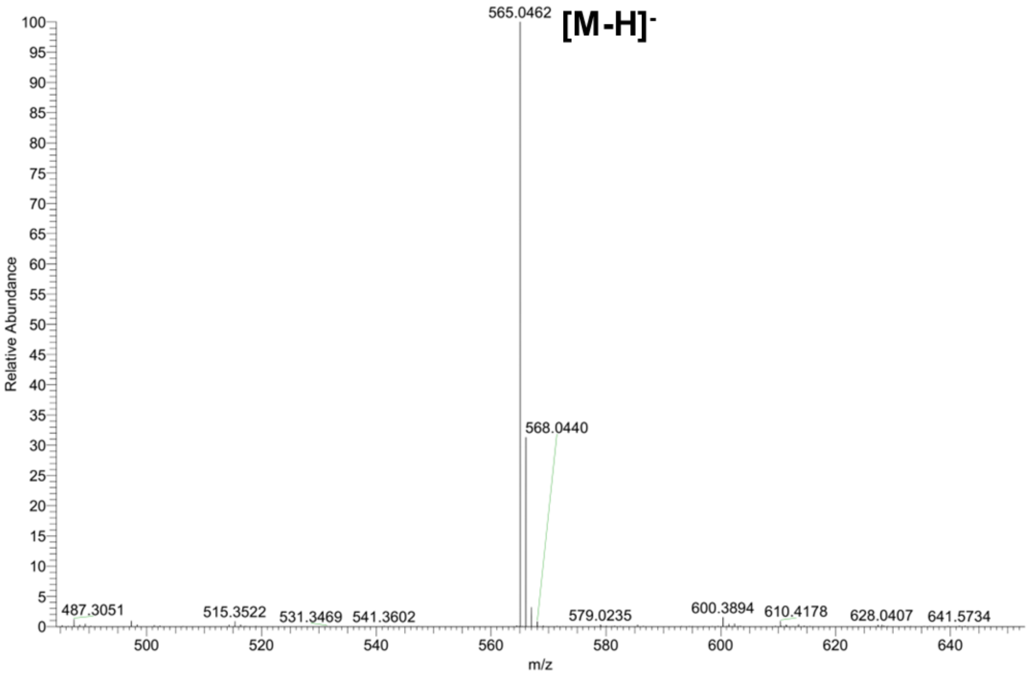

Figure S30: High resolution mass spectrum of **DMS-I** (MeOH, 298 K)

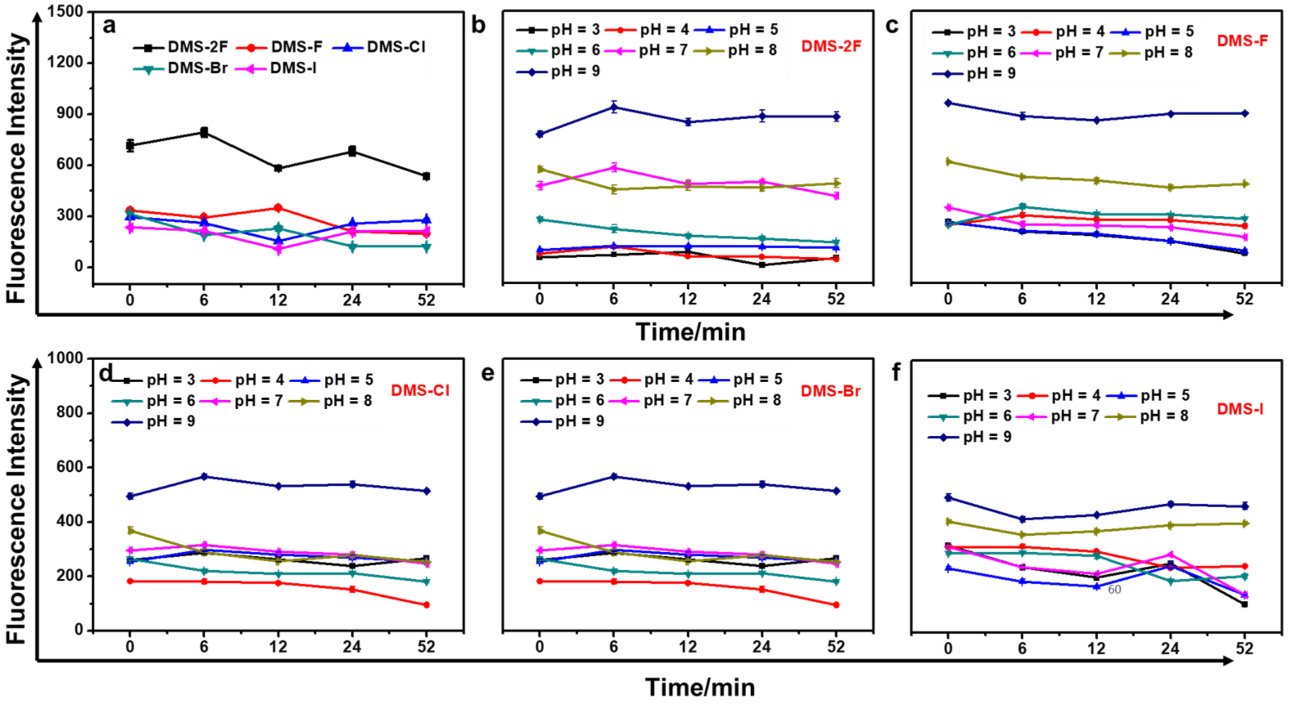

Figure S31: a. The fluorescent stabilities of **DMS-X** (**DMS-F**, **DMS-Cl**, **DMS-Br** and **DMS-I**) during being exposed in 365 nm UV lamp for various time (pH = 7.4, room temperature); and b. **DMS-2F**, c. **DMS-F**, d. **DMS-Cl**, e. **DMS-Br**, f. **DMS-I** in different pH buffer (from 3 to 9) for various time at room temperature (λ_{ex} = 540 nm, λ_{em} = 660 nm, slit = 10/10 nm)

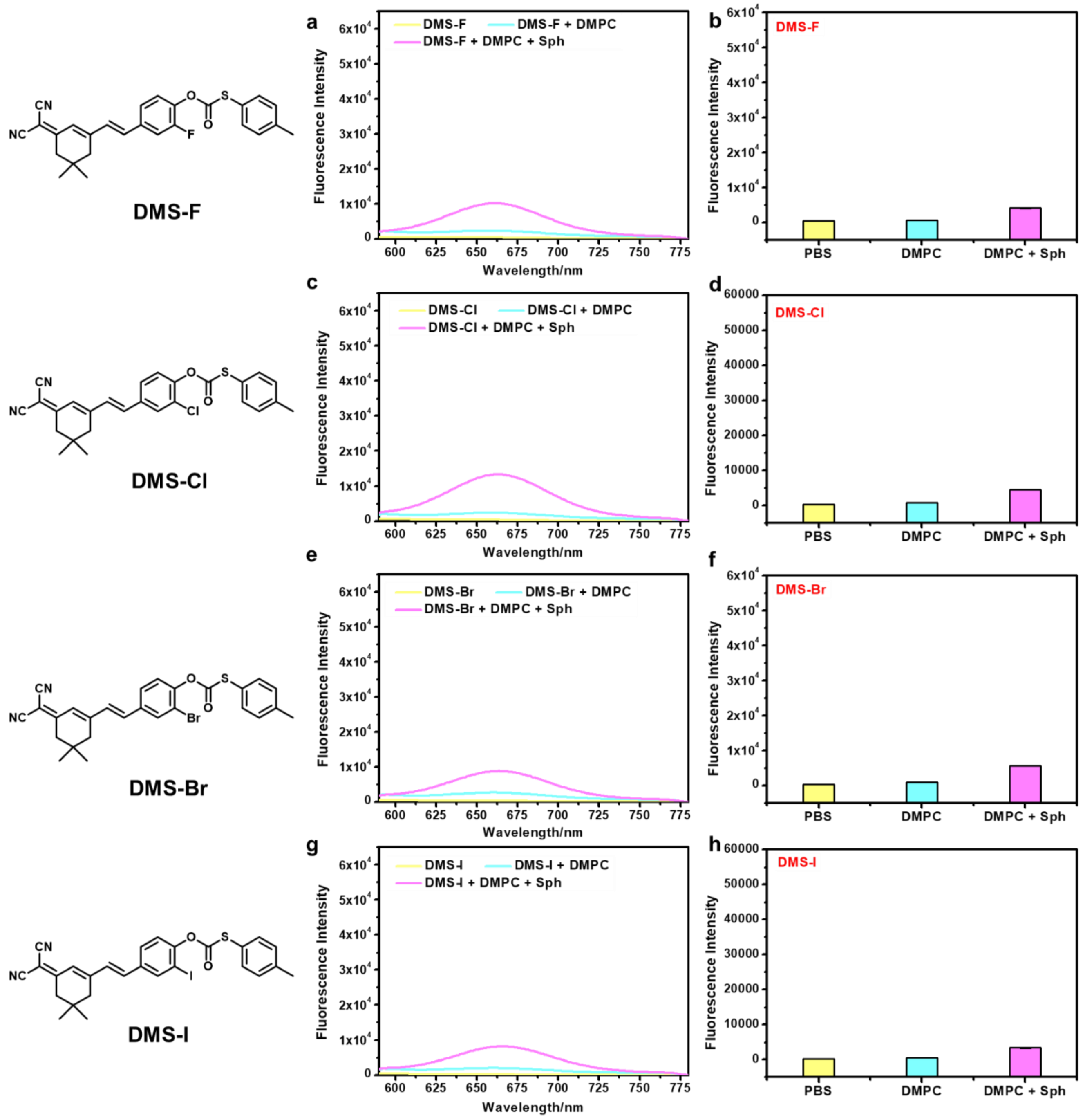

Figure S32: The fluorescent repone results of **DMS-X** (**DMS-F**, **DMS-Cl**, **DMS-Br** and **DMS-I**) for Sph under physiological conditions: a, c, e, g. the fluorescence emission spectra and b, d, f, h. the histograms for fluorescent intensity of **DMS-X**, **DMS-X** in DMPC system and **DMS-X** in DMPC system containing 200 μM Sph, respectively (λ_{ex} = 540 nm, λ_{em} = 660 nm, slit = 10/10 nm, pH = 7.4, 37 °C)

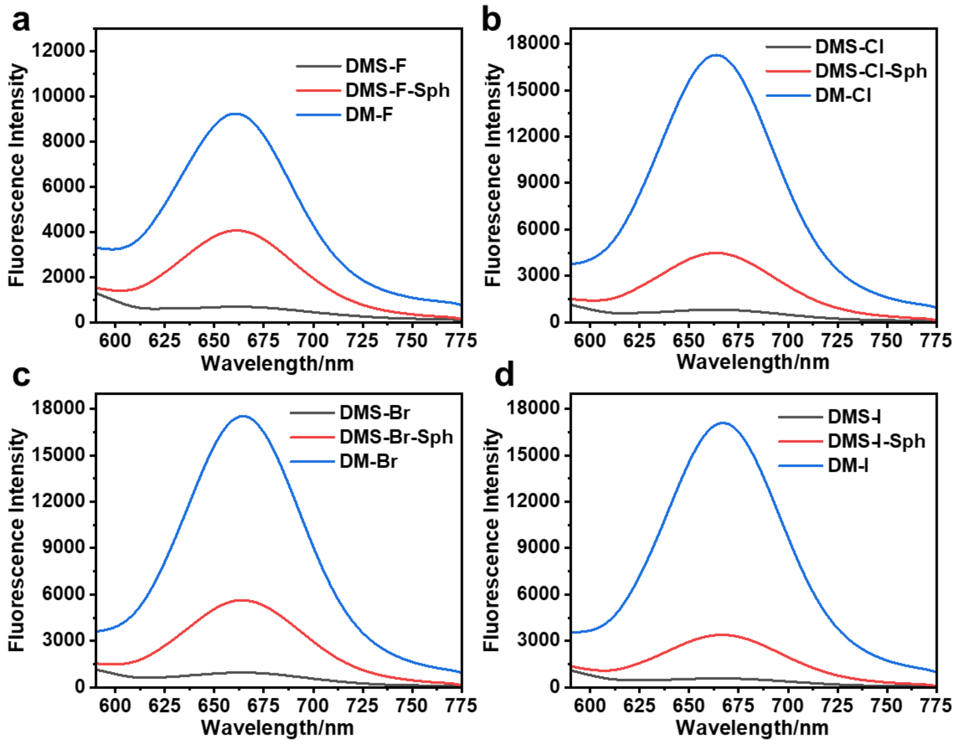

Figure S33: Fluorescence spectra in DMPC vesicles of **DM-X** (X= F, Cl, Br, I) (5 μM), **DMS-X** (X= F, Cl, Br, I) (5 μM) and **DMS-X** (X= F, Cl, Br, I) (5 μM) reacted with Sph (200 μM) (λ_{ex} = 540 nm, slit = 10/10 nm).

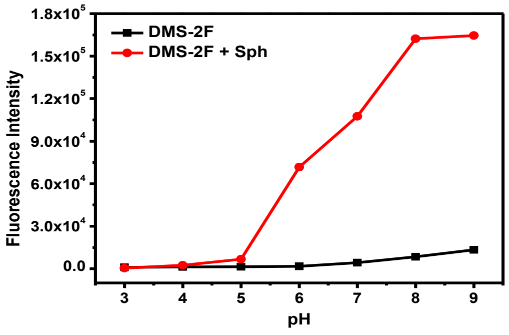

Figure S34: Effect of pH on the fluorescence spectra for **DMS-2F** in response to Sph from 3 to 9 in PBS buffered solution (λ_{ex} = 540 nm, λ_{em} = 660 nm, slit = 10/10 nm).

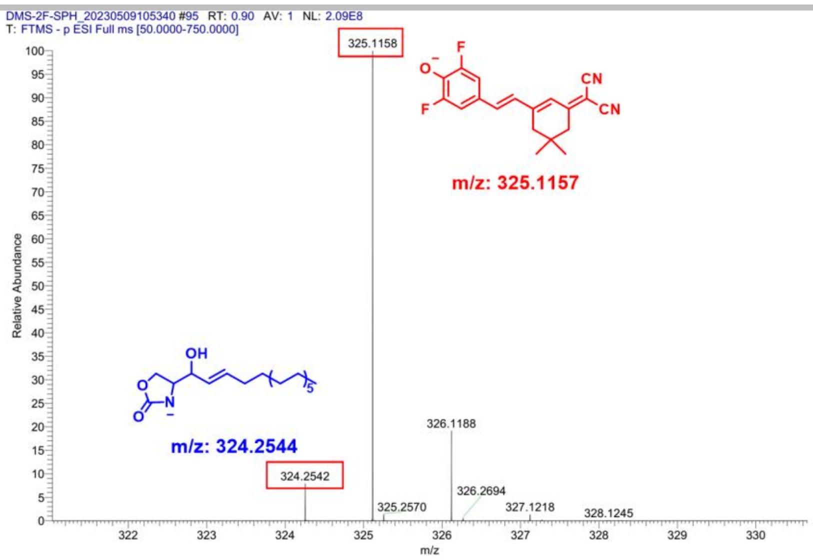

Figure S35: The HR-MS of **DMS-2F** (5 mM) in MeOH after reacting with Sph (100 mM) for 6 h at 37 °C.

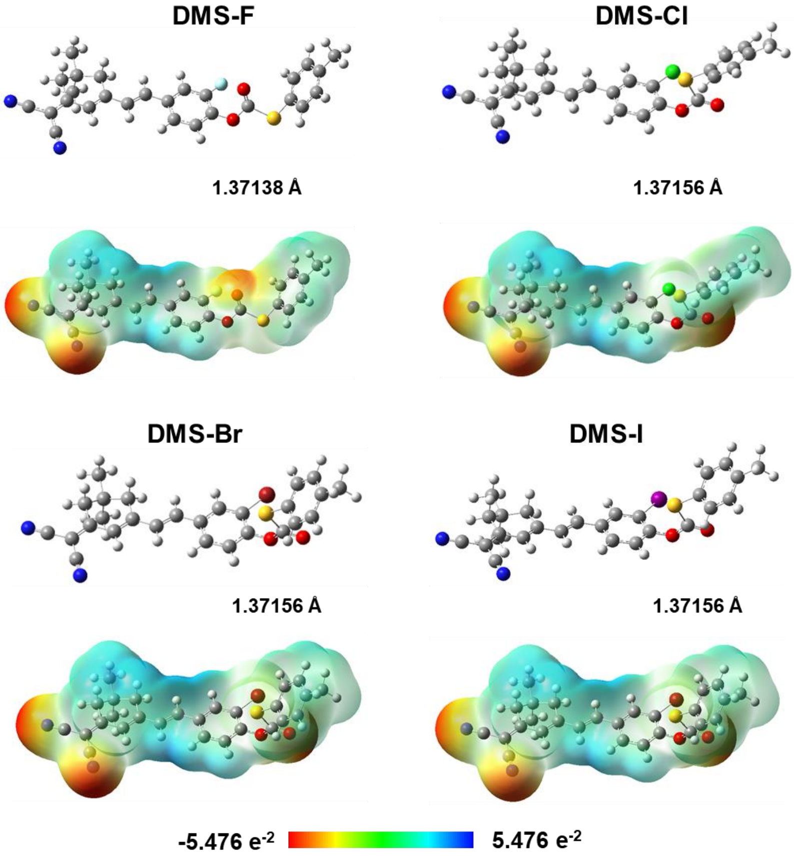

Figure S36: The molecular electrostatic potential (ESP) surfaces and view of the energy-optimized structures for **DMS-F**, **DMS-Cl**, **DMS-Br**, **DMS-Cl**, **DMS-I** from Gauss View.

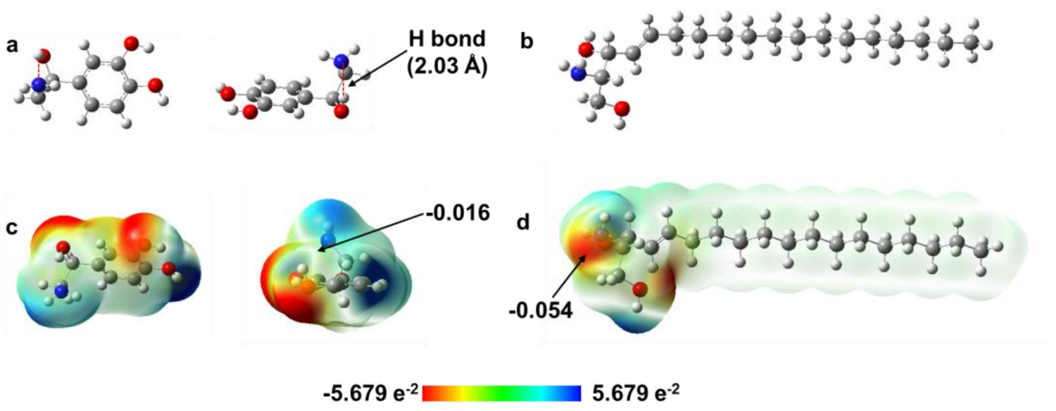

Figure S37. a. The ground-state geometry structure of NE (at the B3LYP/6-31G level); b. The ground-state geometry structure of Sph (at the B3LYP/6-31G level); c. The molecular ESP surfaces of NE. d. The molecular ESP surfaces of Sph.

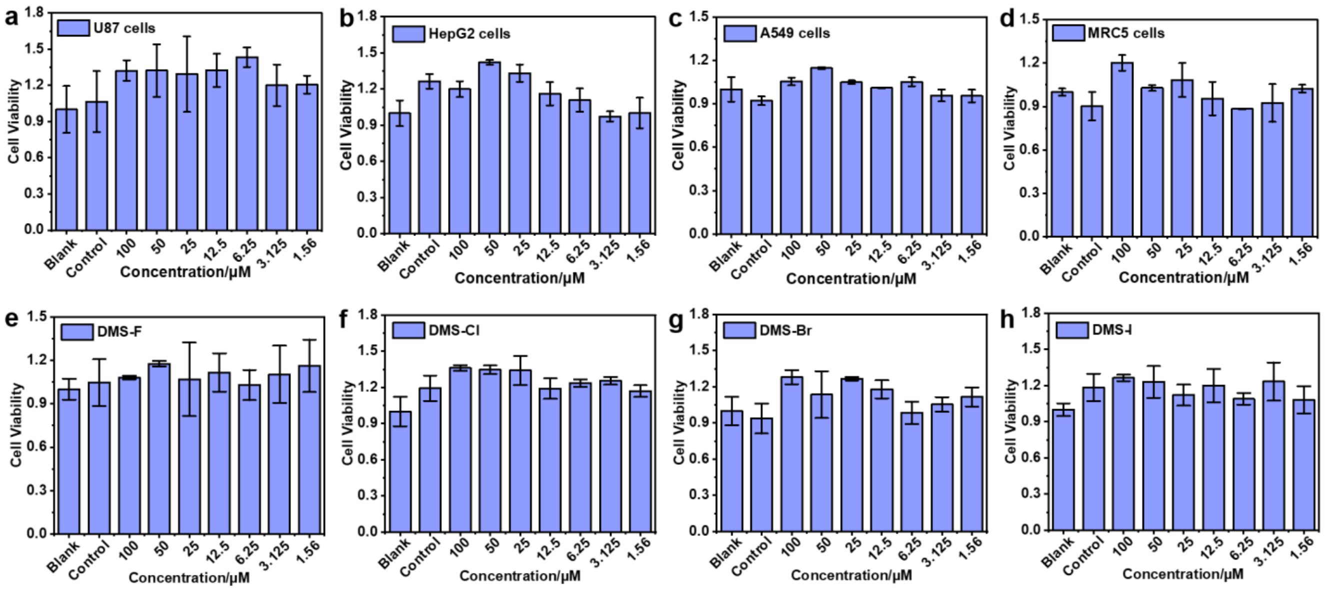

Figure S38: a-d. Cell viability of various cells (U87 cells, HepG2 cells, A549 cells, and MRC5 cells) after incubation with different concentrations of **DMS-2F**; e-f. Cell viability of PC12 cells after incubation with different concentrations of **DMS-X** (e, **DMS-F**, f. **DMS-Cl**, g. **DMS-Br**, h. **DMS-I**). All values are expressed as the mean ± SD of triplicates.

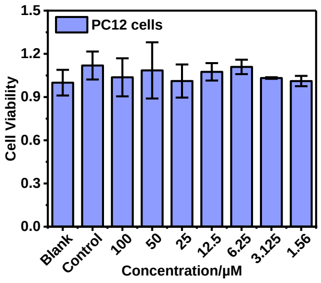

Figure S39: Cell viability of PC12 cells after incubation with different concentrations of **DMS-2F**. All values are expressed as the mean ± SD of triplicates.

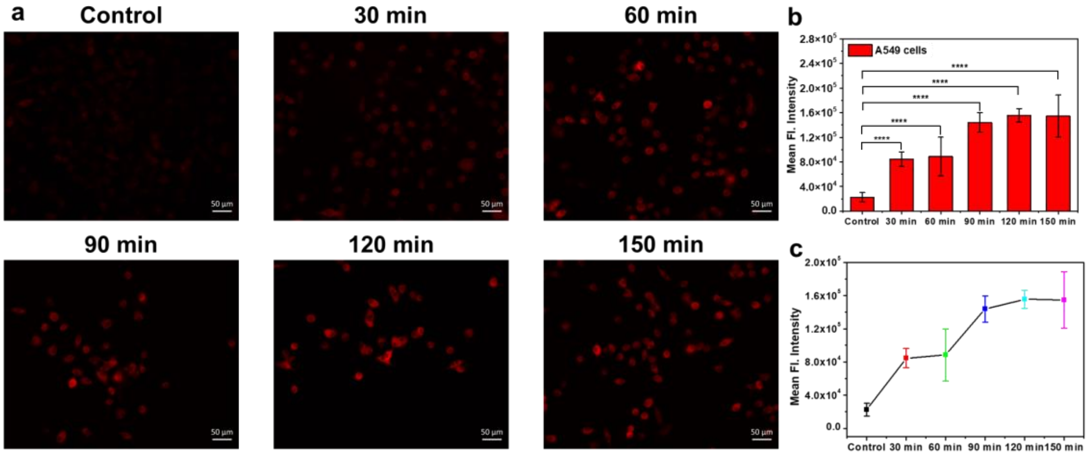

Figure S40: Time-dependent response **DMS-2F** against exogenous Sph addition in A549 cells: a. **DMS-2F**-loaded cells were treated with Sph (40 *μ*M), and the fluorescent images were recorded at different time points; b and c. Mean fluorescent intensities of **DMS-2F**-labeled cells in panel (a). Scale bar: 50 µM. All values are expressed as the mean ± SD of triplicates. One-way ANOVA was used to compare multiple groups: ****P ≤ 0.0001.

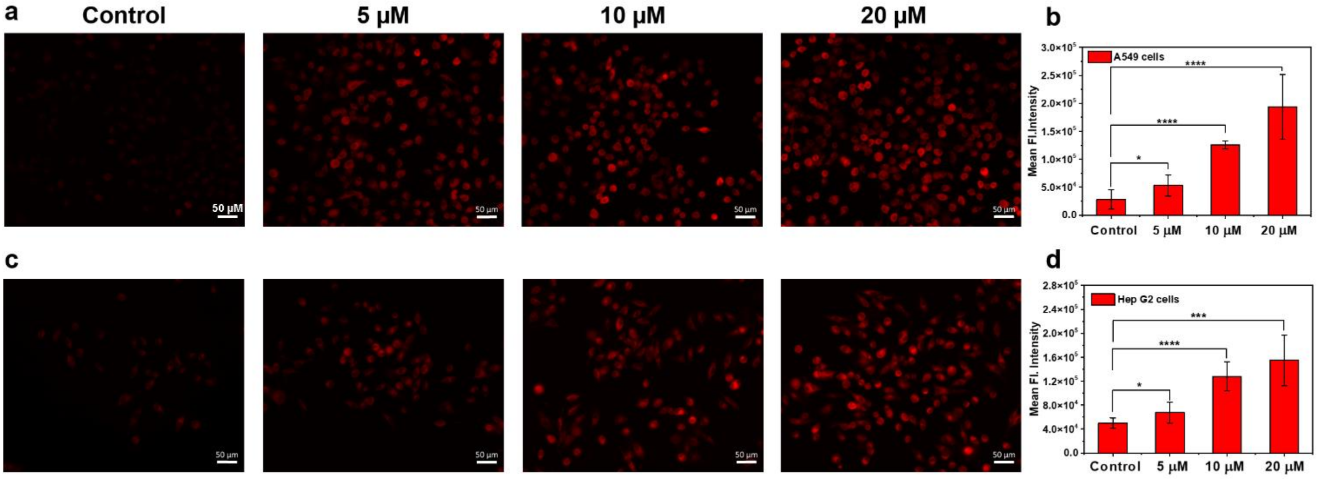

Figure S41. Concentration-dependent response of **DMS-2F** against exogenous Sph addition: a. **DMS-2F**-loaded A549 cells and c. **DMS-2F**-loaded Hep-G2 cells were treated with different concentrations of Sph for 2 h, and then, fluorescent images were taken; b. Mean fluorescent intensities of **DMS-2F**-labeled A549 cells in Panel (a); d. Mean fluorescent intensities of **DMS-2F**-labeled Hep G2 cells in Panel (c). Scale bar: 50 μM. All values are expressed as the mean ± SD of triplicates. One-way ANOVA was used to compare multiple groups: ****P ≤ 0.0001, ***P ≤ 0.001, *P ≤ 0.05.

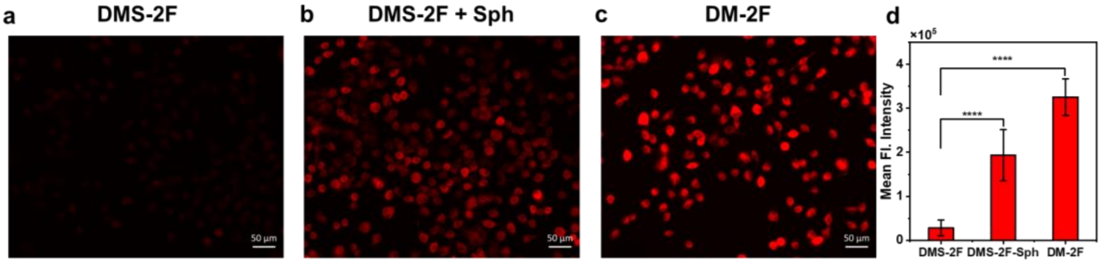

Figure S42: Fluorescence images of A549 cells after a 30 min incubation with a. 5 μM **DMS-2F**, c. 5 μM **DM-2F**; and b. **DMS-2F**-labled A549 cells treated with 20 μM Sph after incubating 2 h; d. Mean fluorescent intensities of A549 cells in panel (a), (b) and (c). Scale bar: 50 μM. All values are expressed as the mean ± SD of triplicates. One-way ANOVA was used to compare multiple groups: ****P ≤ 0.0001.

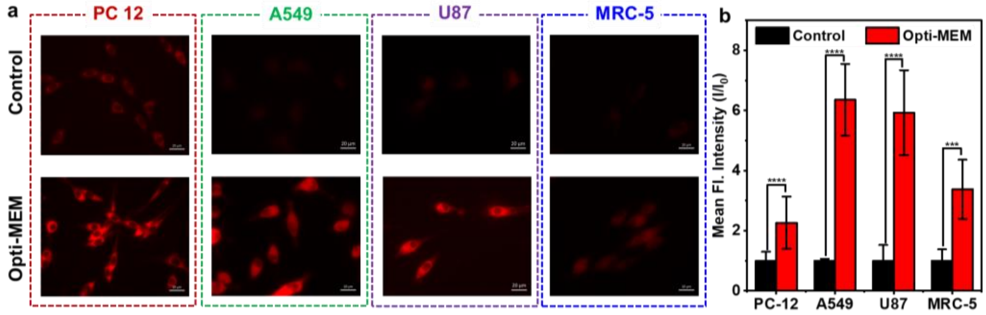

Figure S43. a. Fluorescence images of endogenous sphingosine in living cells: PC12 cells, A549 cells, U87 cells and MRC-5 cells treated with **DMS-2F** for 2 h; b. Mean fluorescence intensities of **DMS-2F**-labeled cells in Panel (a). Scale bar: 20 μM. All values are expressed as the mean ± SD of triplicates. One-way ANOVA was used to compare multiple groups: ****P < 0.0001, ***P < 0.001.

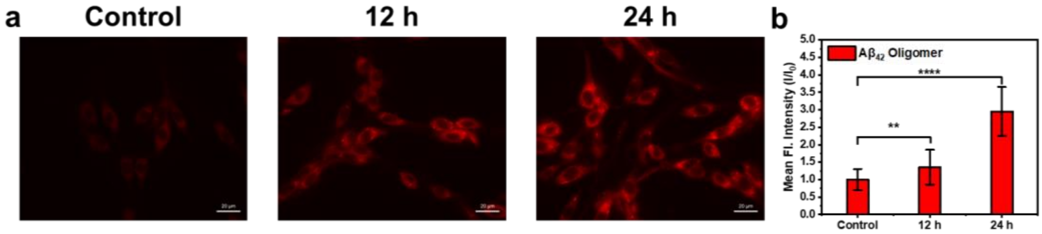

Figure S44. a. Fluorescence images of PC12 cells treated with 10 μM Aβ42 oligomers for different time and then incubated with 10 μM **DMS-2F** for 2 h; b. Mean fluorescent intensities of PC12 cells in panel (a). Scale bar: 20 μM. All values are expressed as the mean ± SD of triplicates. One-way ANOVA was used to compare multiple groups: **P ≤ 0.01, ****P ≤ 0.0001.

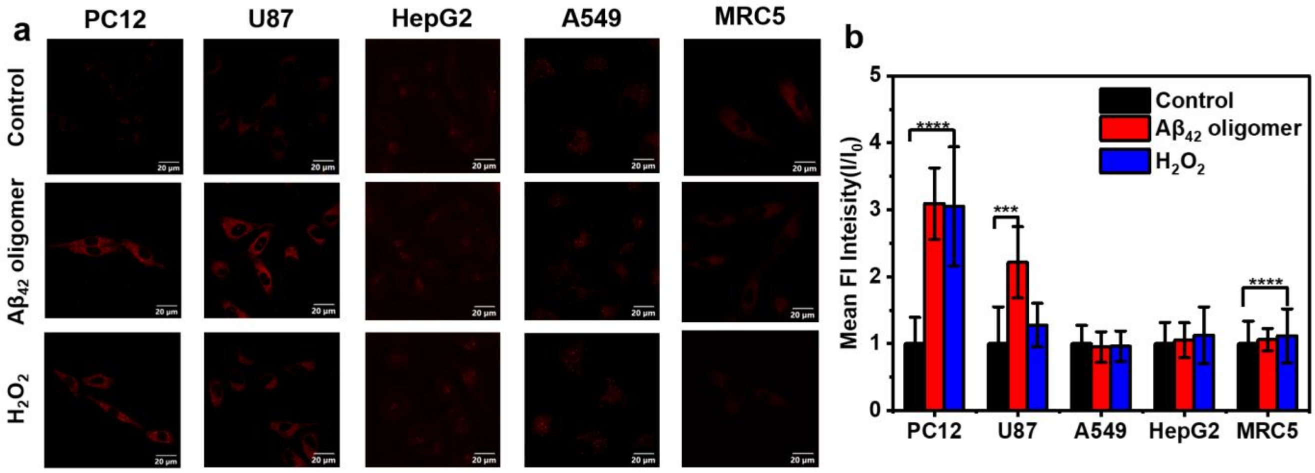

Figure S45. a. Fluorescence images of different cells (PC12 cells, U87 cells, A549 cells, HepG2 cells and MRC5 cells) treated with 10 μM Aβ_{42} oligomers for 12 h and 100 μM H_2O_2 for 4 h and then incubated with 10 μM **DMS-2F** for 2 h. b. Mean fluorescent intensities of different cells in panels (a). Scale bar: 20 μM. All values are expressed as the mean ± SD of triplicates. One-way ANOVA was used to compare multiple groups: ***P ≤ 0.001, ****P ≤ 0.0001.

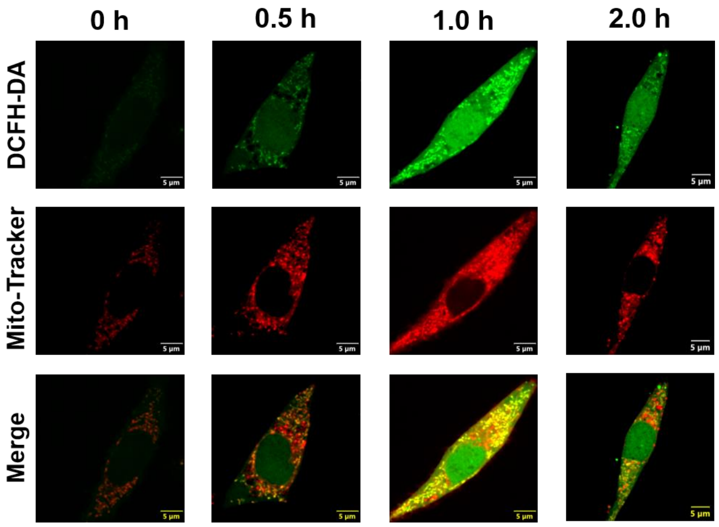

Figure S46. Fluorescence image of PC12 cells induced by Aβ_{42} oligomers at different time to observe the enrichment of ROS (green) in mitochondria (red) and the merge image, scale bar: 5 μM.

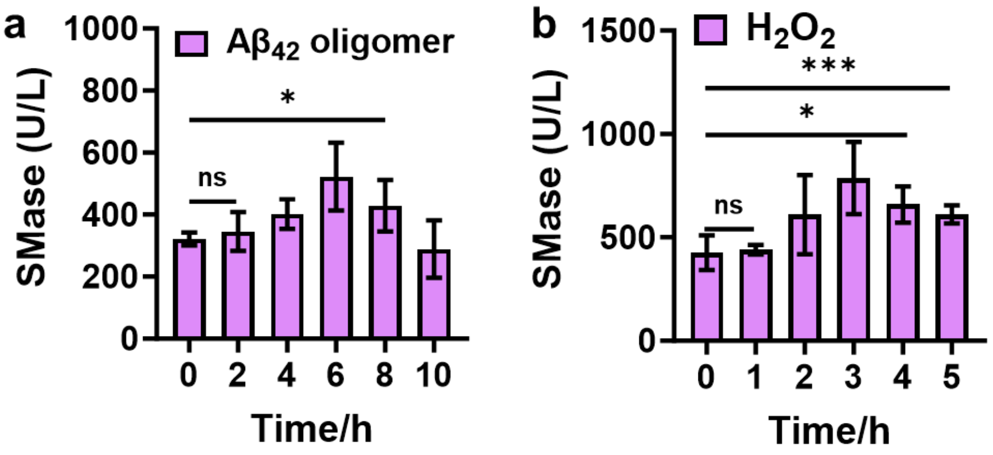

Figure S47. The level of SMase released in the supernatant of PC12 cells treated with Aβ_{42} oligomers and H_2O_2 for different durations measured by ELISA. All values are expressed as the mean ± SD of triplicates. One-way ANOVA was used to compare multiple groups: ns P > 0.05*P ≤ 0.05, ***P ≤ 0.001.

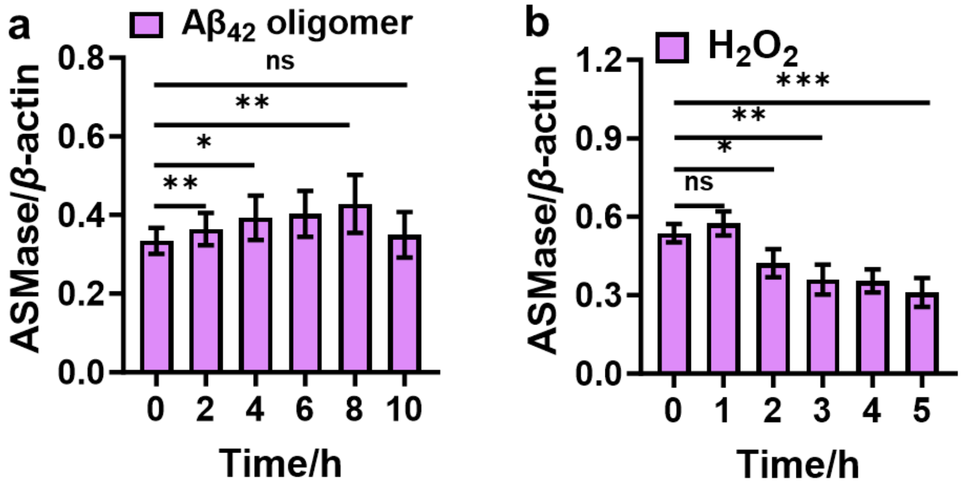

Figure S48. Gray value of the corresponding image of ASMase in Figure 5 (h). All values are expressed as the mean ± SD of triplicates. One-way ANOVA was used to compare multiple groups: ns $P > 0.05$, *$P \le 0.05$, **$P \le 0.01$, ***$P \le 0.001$, ****$P \le 0.0001$.

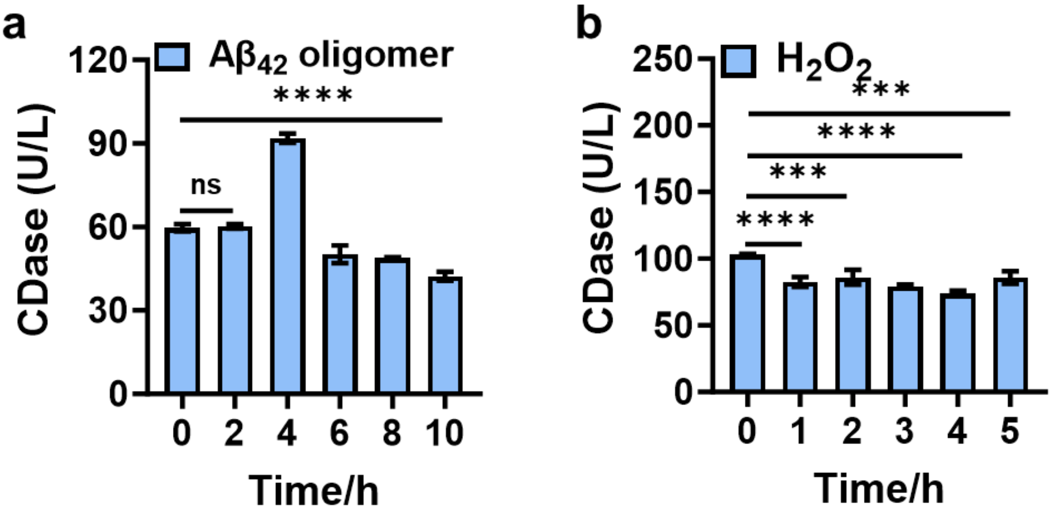

Figure S49. The level of CDase released in the supernatant of PC12 cells treated with $A\beta_{42}$ oligomers and H_2O_2 for different durations measured by ELISA. All values are expressed as the mean ± SD of triplicates. One-way ANOVA was used to compare multiple groups: ns $P > 0.05$, ***$P \le 0.001$, ****$P \le 0.0001$.

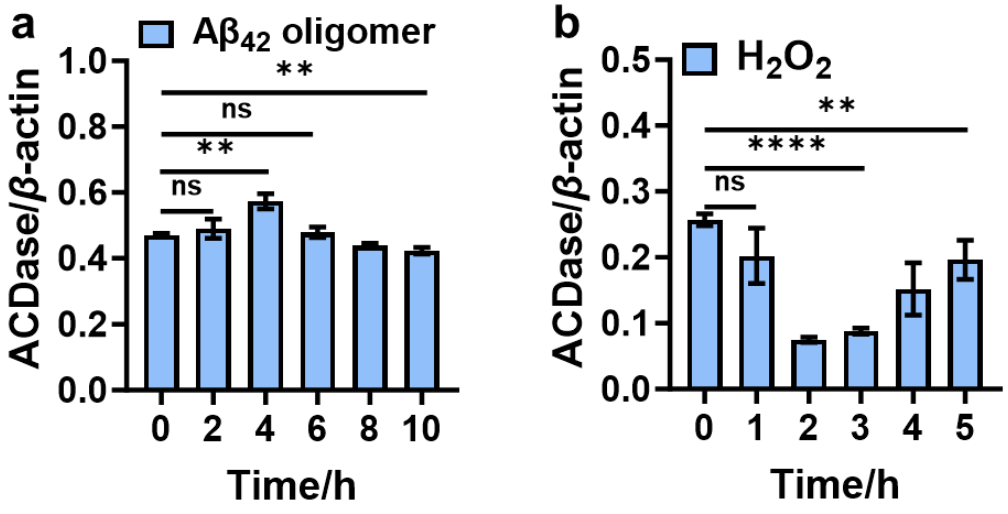

Figure S50. Gray value of the corresponding image of ACDase in Figure 5 (h). All values are expressed as the mean ± SD of triplicates. One-way ANOVA was used to compare multiple groups: ns $P > 0.05$, *$P \le 0.05$, **$P \le 0.01$, ***$P \le 0.001$, ****$P \le 0.0001$.

Reference

[S1]a. Y. Yang, Y. Zhang, M. Ma, H. Liu, K. Ge, C. Zhang, M. Jin, D. Liu, S. Wang, C. Yin, J. Zhang, *Anal. Chem.* **2022**, *94*, 14443-14452; b. H. Yan, Y. Wang, F. Huo, C. Yin, *J. Am. Chem. Soc.* **2023**, *145*, 3229-3237.

Author Contributions

Y.C., Y.Q. and J.Z. conceived the project; Y.C. performed the measurements; Y.C. conducted the experiments; T. H. performed the optical stability assay of probes; J. W. set up the instruments; Y.C. and Y.Q. wrote the paper; Y. C. checked the grammer. J.Z., W.W. and Y.Q. supervised the project. All authors discussed the results and commented on the manuscript.
